# Fine Structural Analysis of Degummed Fibroin Fibers Reveals Its Superior Mechanical Capabilities

**DOI:** 10.1002/cssc.202401148

**Published:** 2024-09-25

**Authors:** D. Eliaz, I. Kellersztein, M. E. Miali, D. Benyamin, O. Brookstein, C. Daraio, H. D. Wagner, U. Raviv, U. Shimanovich

**Affiliations:** ^1^ Department of Molecular Chemistry and Materials Science Weizmann Institute of Science 7610001 Rehovot Israel; ^2^ Division of Engineering and Applied Science California Institute of Technology Pasadena California 91125 USA; ^3^ Institute of Chemistry The Hebrew University of Jerusalem, Edmond J. Safra Campus, Givat Ram 9190401 Jerusalem Israel; ^4^ Present address: Department of Physics of Complex Systems Weizmann Institute of Science 7610001 Rehovot Israel; ^5^ Present address: SilkIt Ltd. Ness Ziona 7403626 Israel

**Keywords:** Silk fibers, Protein self-assembly, *β*-sheet conformation, Mechanical properties, Degumming

## Abstract

*Bombyx mori* silk fibroin fibers constitute a class of protein building blocks capable of functionalization and reprocessing into various material formats. The properties of these fibers are typically affected by the intense thermal treatments needed to remove the sericin gum coating layer. Additionally, their mechanical characteristics are often misinterpreted by assuming the asymmetrical cross‐sectional area (CSA) as a perfect circle. The thermal treatments impact not only the mechanics of the degummed fibroin fibers, but also the structural configuration of the resolubilized protein, thereby limiting the performance of the resulting silk‐based materials. To mitigate these limitations, we explored varying alkali conditions at low temperatures for surface treatment, effectively removing the sericin gum layer while preserving the molecular structure of the fibroin protein, thus, maintaining the hierarchical integrity of the exposed fibroin microfiber core. The precise determination of the initial CSA of the asymmetrical silk fibers led to a comprehensive analysis of their mechanical properties. Our findings indicate that the alkali surface treatment raised the Young′s modulus and tensile strength, by increasing the extent of the fibers’ crystallinity, by approximately 40 % and 50 %, respectively, without compromising their strain. Furthermore, we have shown that this treatment facilitated further production of high‐purity soluble silk protein with rheological and self‐assembly characteristics comparable to those of native silk feedstock, initially stored in the animal′s silk gland. The developed approaches benefits both the development of silk‐based materials with tailored properties and the proper mechanical characterization of asymmetrical fibrous biological materials made of natural building blocks.

## Graphical abstract

The mechanical performance of silk fibroin fibers is typically affected by: 1) thermal degumming, and 2) misinterpretation relying on assumed fibers’ symmetrical cross‐sectional area. The developed low‐temperature alkali degumming preserves fibroin′s structure, improves crystallinity, fibers’ strength and stiffness. This method facilitates the production of high‐purity silk protein with characteristics comparable to native silk protein.

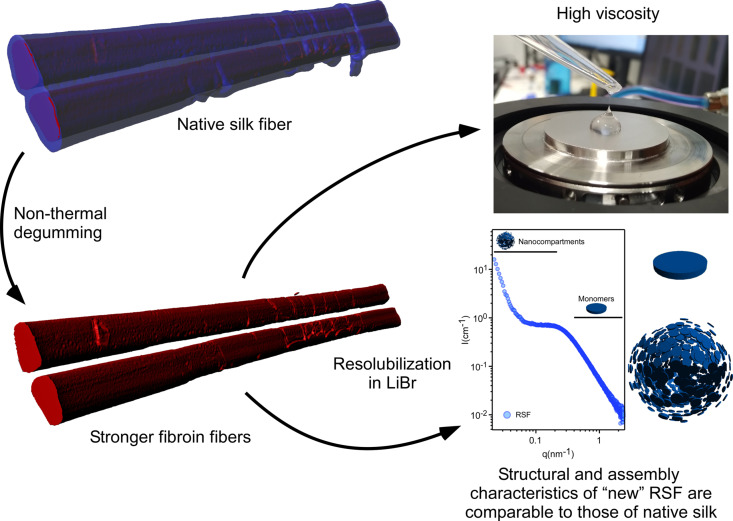

## Introduction

Natural silk cocoon fibroin protein of silkworm, *Bombyx mori* (*B.mori*), is widely used in biomedical applications, in addition to the traditional textile industry. The Food and Drug Administration (FDA) approved *B. mori* fibroin as a versatile biomaterial in the form of films, membranes, gels, sponges, powders, scaffolds, and nanoparticles.[^1^, ^2^, ^3^, ^4^, ^5^] Such popularity is attributed to its exceptional mechanical performance, controllable porosity, good oxygen and water permeability, biodegradability, hemostatic properties, non‐cytotoxicity, low antigenicity, and non‐inflammatory capabilities.[^6^, ^7^, ^8^, ^9^] To benefit from these exceptional features, the fibroin protein core component should be separated from the sericin gum coating layer. The fraction of the sericin layer in *B. mori* silk fibers usually varies from 25 to 30 wt.% of the natural fiber.^[10]^ The core, composed of two adjacent fibroin microfibers, is characterized by an intrinsic hierarchical structural organization.^[11]^ Each of the fibroin microfibers, which are 70–75 wt.% of the silk fiber, is composed of aligned bundles of fibroin nanofibrils. The nanofibrils are made of self‐assembled fibroin protein.^[12]^ The fibroin itself is a large globular protein of approximately 400–450 kDa in size, containing two subunits of heavy and light molecular chains linked via a single disulfide bond.[^13^, ^14^, ^15^] The molecular organization of the protein chains within a single nanofibril alternates between crystalline and disordered regions, where the fraction of the crystalline regions determines the strength and stiffness of the final fiber.^[16]^ The mechanical properties of silk fibers result from the structural transitions of the soluble fibroin protein stored inside the silkworm silk gland (silk feedstock).[^17^, ^18^, ^19^] This transition occurs from a relatively disordered state (a random coil conformation) into a highly ordered *ß*‐sheet‐rich solid fiber due to the molecular flow during the silk fiber′s spinning process from the animal′s gland.[^11^, ^19^, ^20^, ^21^, ^22^, ^23^]

Traditionally, uncoated silk fiber, or degummed fiber, is obtained by removing the sericin layer through a thermal process, during which the native silk fibers are boiled in an aqueous solution of sodium carbonate (Na_2_CO_3_).^[24]^ However, such a procedure damages the fibroin protein component by altering its fold and breaking the disulfide bond between two protein subunits, namely, the heavy and light chains.[^25^, ^26^, ^27^] This damage reduces the mechanical performance of the fibroin fibers, affects their stability, and diminishes their quality compared to the native silk from the silkworm silk gland. These effects are reflected in the poor rheological characteristics and the unpredictable self‐assembling behavior of the reconstituted silk fibroin (RSF) obtained by chemical resolubilization.[^25^, ^27^, ^28^, ^29^]

The CSA of silk and fibroin fibers, as reported in the literature,^[30]^ displays a non‐uniform and asymmetrical shape with numerous defects along the fiber surface. The current literature assumes that both native silkworm fibers and fibroin fibers (degummed fibers) possess a perfect cylindrical shape with diameters of approximately 20 μm and 10 μm, respectively, as commonly measured by scanning electron microscopy (SEM).[^29^, ^31^, ^32^] This assumption leads to a misinterpretation of the asymmetrical geometry of the fiber, thereby leaving its mechanical properties to be elucidated. There has been limited effort in accurately determining the CSA of natural fibers. Previous approaches typically involved embedding and sectioning single fiber samples and imaging using a digital camera, light, or confocal microscopy.[^33^, ^34^] Embedding can obscure the precise perimeter of the fiber sample, particularly in the absence of fluorescence features. Moreover, sectioning can lead to blurred surfaces and distorted images where the transversal CSA of the fiber is visible across different focal planes.^[34]^

In this work, we implemented a simple method for the controlled removal of the sericin layer in an alkaline (NaOH‐based) environment,[^35^, ^36^] without causing thermal damage to the protein fiber core. Our approach preserves the secondary structure and the crystallinity of the fibroin protein component, protects the fiber′s morphology, and enhances the mechanical performance and stability of the fibers, in comparison to untreated silkworm fibers and fibers boiled in a standard Na_2_CO_3_‐containing solution. Furthermore, upon further chemical resolubilization of the fibroin fibers degummed in an alkaline environment, to obtain aqueous protein feedstock, i. e., reconstituted silk fibroin (RSF), the rheological properties of the protein are comparable to those of native silk, stored in the silkworm silk gland. Importantly, since aqueous silk is categorized as a flow‐sensitive material, the rheological characteristics of silk play an important role during the fibers’ spinning process.^[37]^ Consequently, “improving” the rheological characteristics of RSF can potentially facilitate the development of artificial silk‐based materials such as fibers, gels, or films with tailored mechanical properties. Additionally, we propose a new methodology for determining the actual initial CSA of silk fibroin fibers, levering on a 3D reconstruction of standalone silk fibers, thus avoiding potential artifacts from preliminar sample preparation. Accurate determination of the CSA typically forms the basis for calculating the strength of both biological and non‐biological fibers. The developed procedure accounts for the asymmetrical shape of the fibers. Utilizing the two‐parameter Weibull distribution enabled us to detect the presence of defects along the fiber surface morphology, leading to a comprehensive understanding of the mechanical capabilities of fibroin fibers.

## Results and Discussion

### Alkaline, Low‐Themperature Silk Fiber Degumming

**Figure 1:**
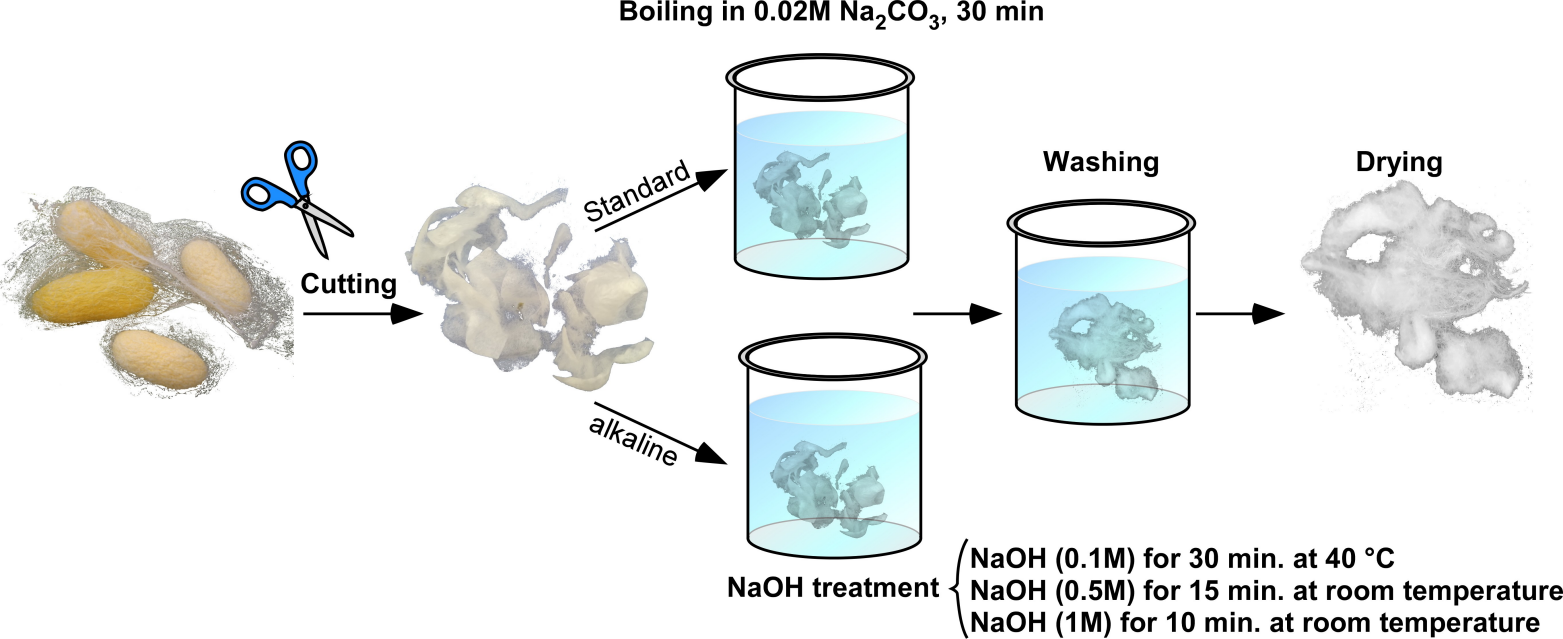
Schematics of the standard and developed silk fibroin fibers degumming. Demonstration of the degumming process. B. mori cocoons were cut into small pieces (below 1 cm). Then, four different treatments were applied for degumming and removal of the sericin glue layer, Na_2_CO_3_, and NaOH (0.1 M, 0.5 M, and 1 M). After the degumming, the fibers were extensively washed with double distilled water and dried at room temperature (see Experimental section).

**Figure 2:**
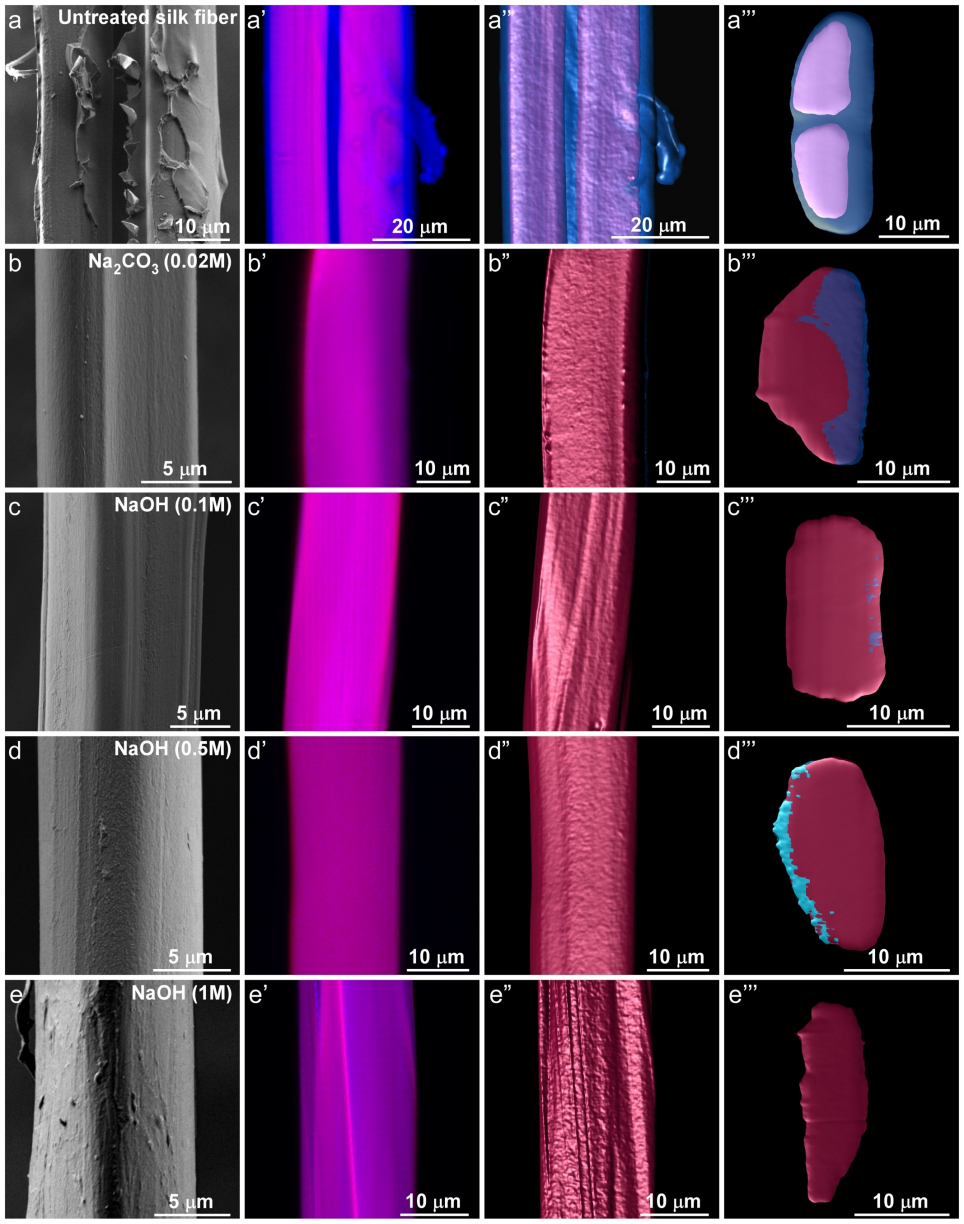
Microscopy analysis of the silk fibroin fibers. (a) SEM image of native (untreated) silk fiber composed of two fibroin fibers coated with the sericin gum layer. (a’–a’’’) Confocal images of the untreated fibers with overlapping intrinsic fluorescence and a fluorescent signal from Nile Red dye, where a’ shows a 2D confocal image and a’’ is a 3D reconstruction of several confocal z‐stacked images; a’’’ represents the 3D reconstruction of a transversal CSA of the fiber. (b) SEM image of silk fibroin fibers degummed by a standard Na_2_CO_3_‐based protocol. b’–b’’’ The corresponding confocal images. (c), (d), and (e) SEM images of silk fibers degummed with 0.1, 0.5, and 1 M NaOH, respectively. c’–c’’’, d’–d’’’, and e’–e’’’ the corresponding confocal images.

The degumming of native silk fibers is an essential step to expose stiff and uniform fibroin core fibers^[10]^ for their further use in the textile industry or for resolubilization into aqueous RSF, which is a basic building block for generating artificial silk‐based materials. Researchers have extensively studied the degumming procedures, including the physical,^[38]^ chemical,[^27^, ^39^] and enzymatic degumming,[^40^, ^41^] where the most efficient and cost‐effective method established is boiling (82–98 °C) native silk fibers (silk fibers containing the sericin glue component) in the presence of aqueous sodium carbonate (Na_2_CO_3_).^[42]^ However, even though Na_2_CO_3_ ‐based degumming formulation is an efficient approach for removing sericin glue, the high‐temperature boiling step induces thermal damage of the fibroin protein, leading to poor fiber mechanical performance, instability of resolubilized protein, and protein composite (heavy and light chain) disintegration. In our attempts to eliminate thermal damage, we degummed native silk fibers in a NaOH‐based alkaline environment, thereby maintained alkaline conditions, preserved the RSF in its soluble state (RSF is soluble at basic pH), and avoided boiling and hence thermal damage. To this end, we incubated native silk fibers for short periods of time (10–30 min) at room temperature in aqueous NaOH solutions at concentrations varying between 0.1 and 1 M (see the scheme of the developed degumming process in Figure 1). We obtained ~100 % yield of sericin gum removal (see Supplementary Table S1), which is ~30 % of the total weight of the composite fiber. Next, we evaluated and compared the impact of the standard (Na_2_CO_3_‐based) vs NaOH‐based degumming approaches on the morphology of fibroin fibers. SEM analysis of untreated native fiber showed the presence of a non‐uniform sericin coating layer (Figure 2a). Unevenness in sericin coating originates from the surface stresses acting on the native fiber during the natural spinning and cocoon handling.[^43^, ^44^] Our SEM analysis revealed that upon degumming of native fiber, either with Na_2_CO_3_ or NaOH, the sericin layer was significantly reduced or was hardly present, as depicted in Figure 2b–e.

Further morphological analysis revealed a geometrical asymmetry in the fibroin fibers, consistent with earlier reports.[^31^, ^45^] Such observation has an implication, on both the interpretation of silk fibers’ morphology and mechanical analysis of the fibers. Traditionally, the ongoing structural and mechanical characterizations of the fibroin fibers encompass both experimental and simulation approaches,[^29^, ^31^, ^32^] which are dependent on an approximation of a cylindrical CSA, thus overlooking their true complexity. Assumptions of a defect‐free longitudinal surface area serve to further complicate an accurate assessment. These simplifications are unsuitable for most biological fibers, including fibroin fibers, and increase the risk of misinterpretations when characterizing asymmetrical fibers.[^29^, ^31^]

To address the existing challenges, we have developed analytical methods with the following objectives: 1) precise differentiation between the sericin coating layer and the fibroin protein core within the composite fiber; 2) quantification of the CSA of asymmetrical composite silk fibers, as well as the CSA of each component within the core‐shell fiber arrangement separately.

To this end, we took advantage of the silk intrinsic fluorescence previously reported by us[^4^, ^46^, ^47^, ^48^] for imaging silk fibers containing both fibroin and sericin^[49]^ (Figure 2a’–e’, and Supplementary Figure S1). Additionally, the Nile red dye, an environmentally sensitive solvatochromic dye, which changes its fluorescence in response to changes in the extinction coefficient of the environment, and thus, the polarity of the material components,^[50]^ has been used for selective staining of the fibroin component (see the Experimental section)[^51^, ^52^, ^53^] with a measured emission peak at 635 nm (Figure 2a’–e’ and Supplementary Figure S1). Combining and overlapping both signals emitted from intrinsically fluorescent regions and regions stained with Nile red dye enabled us to determine the boundaries between the fibroin and sericin components in native silk fiber with high accuracy (Figure 2a’’–e’’ and Figure 2a’’’–e’’’).

Our analysis, based on thousands of z‐stack confocal images (see the Experimental section), revealed the presence of a relatively large amount of sericin in untreated silk fibers (see the regions in silk fibers depicted in Figure 2a’) and either a negligible amount or the lack of sericin in silk fibers degummed either in Na_2_CO_3_ or in NaOH, as shown in Figure 2b’–e’, which is also in good agreement with the SEM analysis (Figure 2a–e).

Next, to determine the CSA of the fibers, we reconstructed confocal z‐stack images using “Imaris” image analysis software and calculated the CSA using a home‐written Matlab script (see the Experimental section and Figure 2a’’–e’’, Figure 2a’’’–e’’’, Supplementary Figures S1 and S2 and Supplementary Code 1).

**Figure 3:**
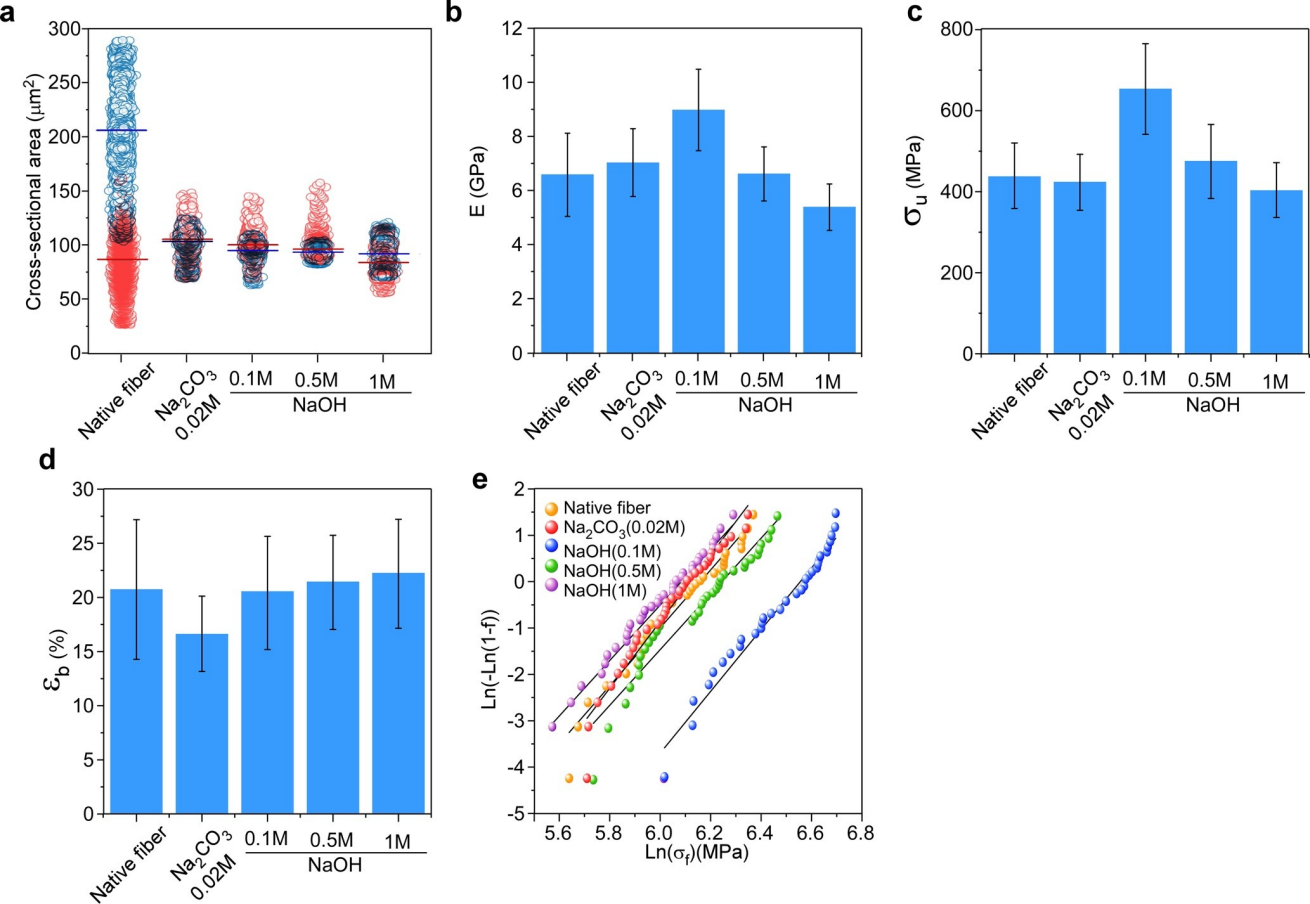
Initial CSA and mechanical properties of the silk fibroin fibers. (a) CSA based on confocal image analysis using a home‐developed MatLab script; (b) Young′s Modulus of native silk fibers and fibers degummed with Na_2_CO_3_, and with 0.1, 0.5, and 1 M NaOH. (c) tensile strength, and (d) strain at the break of the native and degummed silk fibers. (e) Weibull distribution plots showing the strength of the silk fibers. The lines are fits to experimental data, with slopes of (shape parameter), and an intercept of ‐*β*ln(α), where *α* is the scale parameter of the distribution.

Analysis of CSA revealed inconsistencies and a large standard deviation (STD) for silk fibers degummed in the presence of Na_2_CO_3_ (see Supplementary Figure S3a–f and Supplementary Table S2), and the narrowest STD reported for fibers degummed in the presence of 0.5 M NaOH (Supplementary Figure S2a–f and Supplementary Table S2). Such differences most likely originate from better solubility of the sericin component in NaOH or the absence of thermal damage in fibroin protein fibers. A summary of cooperative analysis for the measured CSA of native fibers, fibers degummed via a standard approach, and fibroin fibers treated with NaOH‐based solutions is presented in Figure 3a, Supplementary Figure S3, and Supplementary Table S2. At 1 M NaOH we observed bundle decompositions into separate nanofibrils (see Supplementary Figure S4). The results from the CSA analysis were further used to determine the fiber′s mechanical characteristics (see Table 1), which is described below in detail.

**Table 1:** Summary of the measured tensile data for the native silk fibers and silk fibers degummed using the standard as well as the developed treatment approaches (mean±SD). E – Young′s Modulus; σ_u_ – tensile strength; ε_b_ (%) – strain at the break; *α* and *β* – the scale and shape parameters of the Weibull distribution, respectively ; *n*‐number of tensile measurements.

Fiber sample	E (GPa)	σ_u_ (MPa)	ε_b_ (%)	Weibull parameters on strength		n
				α (MPa)	β	
Untreated silk‐ fiber	6.6±1.5	439.6±83.2	20.6±6.5	474.9	6.2	35
Na_2_CO_3_	7.0±1.	426.0±71.6	16.5±3.4	455.9	7.1	35
NaOH (0.1 M)	9.0±1.5	655.8±113.9	20.5±5.4	702.0	6.6	35
NaOH (0.5 M)	6.6±1.0	477.6±94.0	21.4±4.3	513.5	6.0	35
NaOH (1 M)	5.4±0.9	405.1±69.4	22.2±5.0	440.8	5.9	35

### Mechanical Properties and the Thermal Stability of the Fibroin Fibers

Tensile tests were performed on individual silk fibers, each with multiple repetitions (see the Experimental section), drawn from a population of *B.mori* silk fibers. These fibers were obtained by manually extracting five layers from the cocoon. The primary objective of these tests was to assess the impact of diverse degumming techniques on the mechanical characteristics of the silk fibers. Figure 3b–d displays the Young′s modulus (*E*), tensile strength (*σ_u_*), and strain at break (*ε_b_*) for silk fibers subjected to degumming. The mechanical data parameters were calculated by accounting for the CSA measurements, as explained earlier, both before and after the degumming procedures. This information is depicted in Figure 3a and Supplementary Figures S2 and S3. These measured and computed mechanical properties play a critical role in evaluating the effectiveness of different degumming treatments on the structural behavior of silk fibers, thereby contributing to potential applications of silk as a reinforcing material.

Incorporating the initial actual CSA of the fibers, as shown in Figure 3a, into the mechanical calculations is imperative for achieving precise understanding of the mechanical properties of these fibers.^[54]^ Furthermore, given the significant plastic deformation observed in silk, along with the associated mechanical phenomena, such as strain hardening, we find it essential to determine the true stress and strain of silk fibers. These values are of utmost importance in comprehending the genuine mechanical characteristics of the material. A thorough mathematical description of the true stress and strain values of the fibers is provided in Supplementary Note S1.

The true tensile stress‐strain curves, computed using Supplementary Equations (E5) and (E6), exhibit an initial elastic response (∼1–1.5 % strain), followed by a prolonged plastic behavior in all fibers (Supplementary Figure S5). The observed high scattering in these curves (Supplementary Figure S5) is a characteristic trait common in imperfect biological materials[^32^, ^55^] characterized by low symmetry and the presence of defects. Upon yielding to applied stress, the fibers display a strain‐hardening behavior, accompanied by stress fluctuations as they progressively fail. Similar strain hardening phenomena have been previously observed in spider silk^[56]^ and *A. perni* silk,^[57]^ attributed to the opening, rearrangement, and gradual breaking of *β*‐sheets within the fibroin fibers during deformation along the fiber axis.^[57]^ Furthermore, strain‐hardening helps in resisting failure and enhancing the fiber′s mechanical strength, as seen in other biological materials with similar capabilities.^[58]^

At its highest levels of hierarchy, untreated *B.mori* silk fibers consist of two fibroin microfibers enclosed within a sericin shell, as shown in Figure 2a. During the tensile test, the mechanical response of each fibroin fiber was observed, as indicated by arrows in Supplementary Figure S5a. As strain propagated throughout the stretching process, the applied stress on the fiber progressively increased. When the first fibroin fiber failed, an appreciable drop in stress magnitude, approximately half, was observed. Subsequently, the strain continued to increase until the catastrophic failure of the second fibroin fiber was eventually reached.

The elastic modulus, strength, and strain at the break of *B. mori*, obtained from our measurements (Figure 3b–d), are comparable with the values reported in the literature.[^32^, ^59^, ^60^] However, minor discrepancies, particularly in σ_u_, appear to stem from variations in factors such as the CSA used in the calculations, variable conditions during silk harvesting, and mechanical testing.[^32^, ^60^] When compared to the mechanical properties of native fibers, the standard degumming method (boiling in Na_2_CO_3_) results in slightly higher modulus values, with only an 8 % increment (6.64 GPa and 7.08 GPa, respectively, as shown in Figure 3b). Concurrently, it slightly affects both strength and strain at break, which show values of ∼426 MPa for strength and ∼17 % for strain at break (Figure 3c–d). These findings suggest that the boiling process introduces more defects along the surface of the fibroin fibers, potentially compromising the structural integrity of the *β*‐sheets within the fibers, thus reducing their strength and strain capabilities.

Degumming silk fibers using low molar ratios of NaOH (up to 0.5 M) yielded a synergistic mechanical effect, with improvements or negligible harm to the modulus, tensile strength, and strain at break of the fibers, as depicted in Figure 3b–d, compared with both untreated and Na_2_CO_3_‐degummed fibers. Among these methods, the most efficient one was found to be the treatment with 0.1 M NaOH. Compared with the standard degumming process, silk fiber treated with 0.1 M NaOH resulted in an enhanced modulus and strength of 27.5 % and 54 %, respectively. Furthermore, the strain at break was improved by 23.7 %. To validate our findings, we conducted a one‐way ANOVA test (Supplementary Figure S6) that indicated significant differences in modulus and strength between the fibers treated with 0.1 M NaOH and the other fiber samples (i. e., p<0.05). Compared with other degummed silks reported in the literature, degumming *B.mori* silk with 0.1 M NaOH yields higher strength than degummed silk from *A.mylitta*, *A.pernyi*, *S.c.ricini*, and other silks, with improvements ranging from 30 % to 130 %.[^32^, ^59^, ^60^] Similarly, the elastic modulus of *B.mori* silk fibers treated with 0.1 M NaOH shows between 70 and 126 % enhancement, compared to other silk fibers.^[60]^ However, treatment with 1 M NaOH resulted in fibers with lower values of E and σ_u_ (Figure 3b–d), likely due to the harsh degumming process, which damaged the surface and the structural integrity of the fibroin fibers (Figure 3e). However, this aggressive degumming process also induced larger deformations.

Fiber strength is influenced by both defects along the fibers and the non‐uniform CSA. To gain a deeper understanding of and correlate the effect of the degumming method on fiber strength, we employed a two‐parameter Weibull distribution^[61]^ whose rationale is detailed in Supplementary Note S2. Initially, the data were ranked, with each data point being associated with a failure probability. Subsequently, these data were plotted using Supplementary Equation (E9), resulting in Figure 3e. We observed that silk degummed with the standard method (Na_2_CO_3_) exhibited a higher *β‐*value (shape parameter), as shown in Figure 3e and in Table 1, indicating lower variability in strength compared with the 0.1 and 0.5 M NaOH degumming treatments, which displayed wider strength distributions, along with lower *β‐*values. The shape parameter values found in this work are higher than those reported in the literature, suggesting the homogeneity of our developed degumming processes.[^62^, ^63^] The characteristic strength, represented by the scale parameter (*α*), shown for each sample in Table 1, specifies the distribution′s location along the x‐axis, which is close to the average strength of such a fiber.

**Figure 4:**
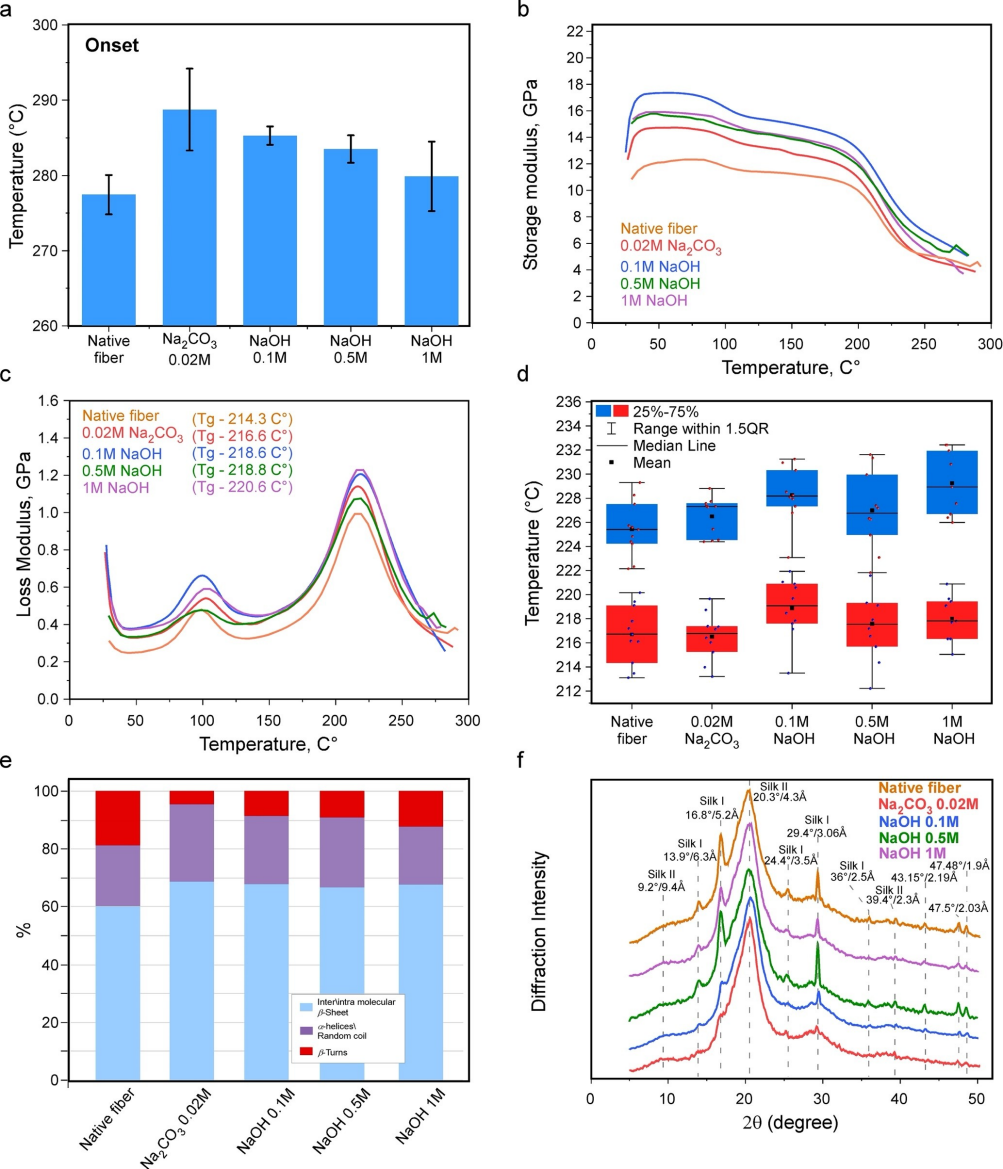
Dynamic mechanical properties, thermal stability, and diffraction pattern of the silk fibers. (a) TGA analysis for the fibers before and after different degumming treatments. () (b) Storage and (c) loss modulus for the fibers before and after different degumming treatments. (d) The glass transition temperature of degummed fibers measured from tan(delta) (blue boxes) and from the loss modulus (red boxes). (e) Comparative analysis of the secondary structure of the Amide I FTIR spectra of degummed fibers. The band positions of the *β*‐sheets at 1610–1635 cm^−1^, anti‐parallel *β*‐sheets at 1690–1705 cm^−1^, random coil and *α*‐helixes at 1635–1665 cm^−1^, and *β*‐turns at 1665–1690 cm^−1^. (f) XRD analysis was conducted on degummed fibers and compared with native silk fibroin fibers. Seven main peaks were recorded at 2θ of 10°,13°,18°,20.7°,25°, and 29°.

The differences in the degumming treatments, in particular, with (standard protocol) and without (NaOH‐based degumming) heat‐induced degradation, might lead to variations in the thermal stability of the fibers. Thus, for example, according to previous reports, the fibers containing a higher degree of molecular orientation as well as a higher degree of crystallinity tend to show higher thermal stability.[^27^, ^64^, ^65^] To this end, we performed a thermogravimetric analysis (TGA), which is summarized in Figure 4a and Supplementary Figure S7. The data obtained from the TGA analysis have been processed by using two distinct approaches: Onset (Figure 4a), which is the intersection between the baseline and the tangent at the point of the highest slope, corresponding to the starting point of the heat‐induced material loss. The second approach involves deriving the weight against the temperature (Supplementary Figure S7), which is the maximum of the material loss. In agreement with previous investigations,[^27^, ^64^, ^65^] our results showed three major peaks of weight loss for untreated fibers and fibers subjected to different degumming treatments. The 1^st^ peak, varying between 40 and 50 °C (weight loss of 6–8 %), is associated with a loss of moisture; a 2^nd^ peak between 270 and 300 °C, is associated with the slow thermal decomposition stage (weight loss 40–45 %), and a final weight loss at the third stage was recorded between 300 and 350 °C (weight loss of 19–22 %), caused mainly by the breakage of peptide bonds and side groups. Interestingly, although only small differences have been detected for the thermal stability of the silk fibers (see Supplementary Figure S7 and Supplementary Table S3) degummed by using the standard protocol and NaOH‐based treatment, the major differences were in the reproducibility of the measured values. Thus, for the standard protocol, large variations in weight loss temperatures have been recorded (STD<~2.6 %), which most likely originates from many defects caused by thermal treatment. The TGA data recorded from samples degummed in the presence of NaOH were highly reproducible with STD<~6 %.

To better understand the influence of the degree of crystallinity and molecular orientation on the thermal and mechanical properties of the fibers, we conducted additional dynamic mechanical analyses on individual fibers, as illustrated in Figures 4b–c and in Supplementary Figure S8. Consistent with the quasi‐static mechanical tests presented in Figure 3b, fibers degummed with 0.1 M NaOH exhibited higher storage modulus values (Figure 4b). In general, it is evident that degumming silk fibers with NaOH results in higher storage modulus values compared with removing the sericin coating layer with Na_2_CO_3_. This difference can be attributed to the potential thermal degradation induced during the degumming process. As the temperature increases, an initial decrease in the storage modulus can be observed at ∼100 °C, attributed to the evaporation of water molecules attached to the fibroin microstructure. This observation is supported by the initial peak in the loss modulus shown in Figure 4c. The secondary degradation of mechanical properties occurs after reaching the glass transition temperature (Tg), determined from the main peak in the loss modulus plot (Figure 4c). As the temperature approaches the Tg value of the fiber, the transition from a glassy behavior to a rubbery behavior becomes apparent. Given the crystalline morphology of the fibers, the polymeric fibroin chains become immobilized, resulting in the fiber maintaining a certain level of storage modulus. A higher storage modulus observed after passing the Tg value may be correlated with a greater degree of crystallinity in the fiber (Figure 4b). Higher Tg values were recorded for fibers treated with NaOH, compared with those treated with the standard thermal protocol (Figure 4d), suggesting that degumming the fibers with NaOH results in a higher degree of crystallinity in the fiber. Furthermore, it is possible that the molecular arrangement after degumming with NaOH induced a preferred molecular orientation within the fibroin fibers, thereby enhanced the Tg and the storage modulus of the material.

### The Structural Characteristics and the Mechanism Underlying Silk Fiber Degumming

To better understand the structural origin of the increased mechanical strength for NaOH‐treated silk fibers, compared with standard treatment, we performed a Fourier transform infrared spectroscopy (FT‐IR) analysis in which we evaluated the changes in the silk protein secondary structure. In general, the vibrational spectra of proteins/peptides are characterized by two major bands, namely, amide I (1600–1700 cm^−1^) and amide II (1480–1600 cm^−1^), which correspond to C=O and NH bend/CH stretching, respectively. The results of the FTIR analysis, summarized in Figure 4e and Supplementary Figure S9 highlight the existence of rather minor variations in the secondary structure content of the silk fibers degummed under different conditions. Thus, fibers degummed by Na_2_CO_3_ contained 27 % of *α*‐helix/ random coil content and 4.5 % of *β*‐turn, whereas fibers degummed with NaOH showed 20–24 % of *α*‐helix/ random coil content and 8.7–12.4 % of *β*‐turns, respectively. The level of *β*‐sheets in all degummed fibers varied between 66.7 and 68.7 % (Figure 4e and Supplementary Figure S9). Thus, overall, we observed a slightly higher content of *α*‐helix, random coil, and *β*‐turn content in fibers degummed using the standard protocol vs. fibers degummed by NaOH.

We also performed X‐ray analysis to probe the differences in the crystalline fraction of the treated fibers as well as gel electrophoresis to examine the integrity of the protein molecules. Generally, silk fibroin protein composing fibroin fibers mainly occurs in crystalline or amorphous (random coil) form. The fine balance between these two forms – a large fraction of the crystalline component decorated with a small fraction of disordered regions, highlights the exceptional mechanical characteristics of the fibers. Our XRD analysis revealed the characteristic diffraction peaks of 2θ at 9.5°, 20.7°, 24.3°, and 39.7° (the corresponding crystalline spaces are 9.2, 4.3, 3.5, and 2.3 Å,[^66^, ^67^] as depicted in Figure 4f) indicting the Silk II structure, which is a *β*‐sheet crystalline form, whereas diffraction peaks of 2θ at 12.2°, 19.7°, and 24.7° (the corresponding crystalline spaces are 6.3, 3.67, and 3.5 Å[^66^, ^68^, ^69^]) indicate a Silk I structure, which is a disordered random coil silk protein. Interestingly, the diffraction peaks of 2.5, 3.06, 3.5, 5.2, and 6.3 Å were absent in Na_2_CO_3_‐treated samples, indicating a reduction in both the crystalline and disordered fractions of the protein. We hypothesize that such an event might originate from the loss/disintegration of the protein subunits.

**Figure 5:**
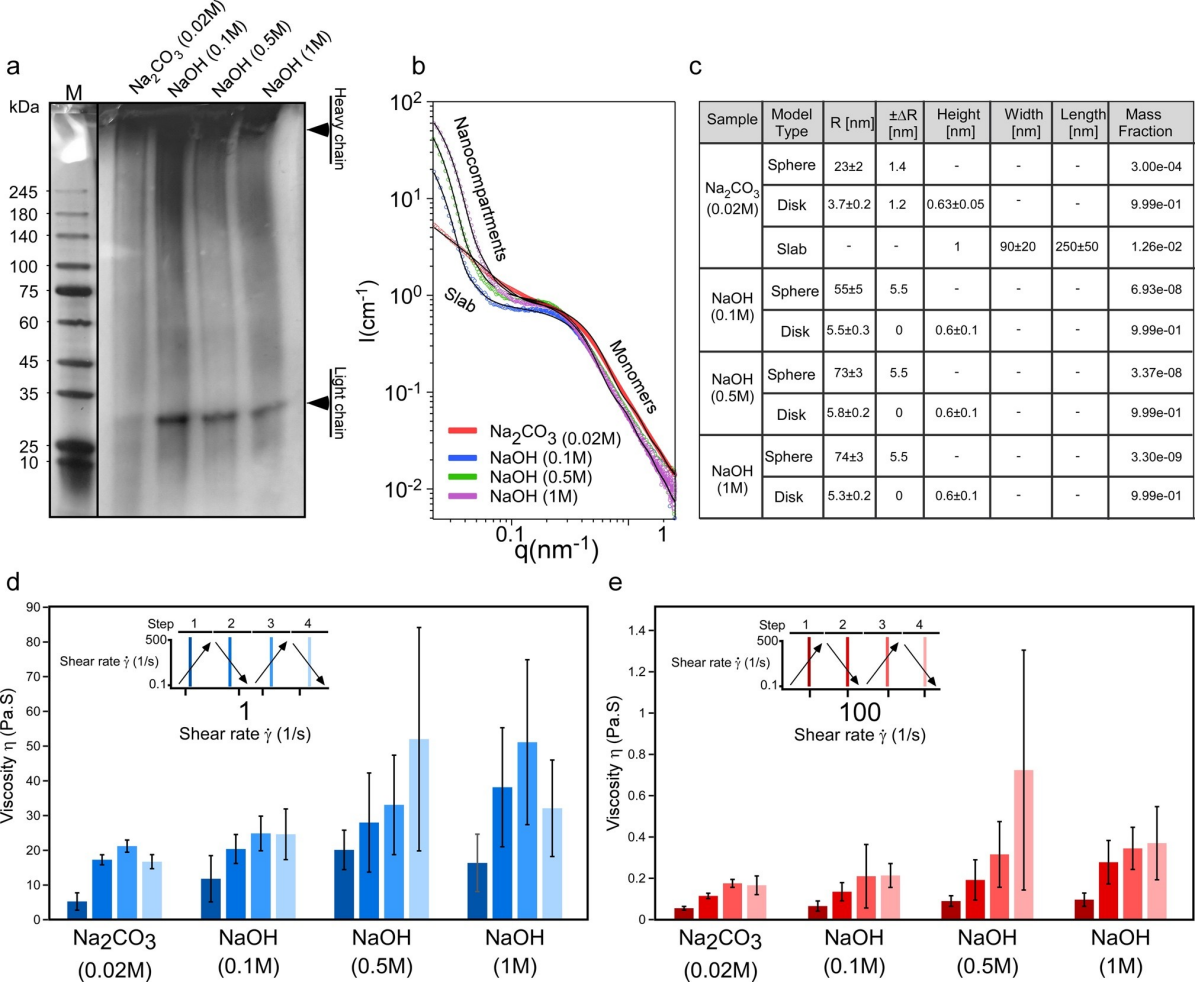
Structural characteristics and the mechanism underlying soluble silk fibroin. (a) Gel electrophoresis analysis of silk fibroin protein molecules, (b–c) SAXS analysis of soluble silk fibroin. (b) Azimuthally integrated background‐subtracted solution X‐ray scattering absolute intensity, I, as a function of the magnitude of the scattering vector, q, from native silk and soluble RSF, obtained from the different degummed approaches. (c) The combination of models that were used to fit the SAXS data. The table shows the model type (Sphere, Disk, or Slab), the radii of the model, R, the polydispersity in the radii (ΔR), the height of the disks or slab, and the mass fractions of protein in each model. (d–e) Rheology analysis of changes in viscosity in response to applied shear, with increasing shear rates from 0.1 to 500 s^−1^ and back from 500 to 0.1 s^−1^ twice. The following samples were analyzed: RSF solution obtained via chemical resolubilization of the silk fibers degummed in the presence of Na_2_CO_3_, and 0.5 M NaOH. The graph demonstrates the viscosity and shear rate of 1 (d) and 100 (e), with their standard deviations (mean±sd, n≥3).

To further examine the integrity of the fibroin protein molecule as a function of applied treatment, an electrophoretic protein gel analysis was performed. More specifically, a denaturing gel was used to test the presence of heavy and light chain protein subunits. *β*‐mercaptoethanol, a chemical used in electrophoretic gel analysis, can break, via a reduction reaction, disulfide (S−S) bridges, and denature protein, providing additional information about the protein chain. The results, which are summarized in Figure 5a, revealed an absence of a light chain subunit in samples degummed by using a standard Na_2_CO_3_‐based protocol, whereas for NaOH‐treated samples the presence of both heavy and light chain protein subunits was observed. Such an observation validates the NaOH‐based degumming method for gently removing the gum component without damaging the protein subunits.

We also performed small angle x‐ray diffraction (SAXS) analysis of soluble silk fibroin from the standard Na_2_CO_3_‐based protocol and compared with the NaOH‐treated samples (see the chemical resolubilization procedure in the Experimental section). Generally, resolubilized silk fibroin (RSF) refers to the soluble form of silk fibroin and can potentially be further processed into different material formats. The efficiency and reproducibility of RSF processing rely on the initial quality of the RSF protein, which is expected to exhibit similar, or ideally identical, behavior to the native silk protein stored inside the silkworm silk gland. The “desired behavior” includes the stability of RSF in soluble form (which is essential for the pre‐processing step), and the rheological characteristics, especially the response to shear forces, which is important for controlled RSF spinning and/or other types of processing. The correct balance between these two parameters enables the processing of RSF into materials with tailored properties, such as controlling its mechanical strength, degradation rate, and biocompatibility, making it suitable for a wide range of applications. In the context of stability parameters, we have recently found and reported[^12^, ^19^] that silk fibroin tends to self‐stabilize via the formation of spherical structures, termed compartments, in which the structural transitions are precisely regulated. Therefore, we next tested and compared the propensity of compartmentalization in RSF obtained from fibers degummed via thermal treatment (the standard Na_2_CO_3_‐based protocol) and via NaOH‐based degumming. Our initial atomic force microscopy (AFM) analysis revealed that silk fibroin resolubilized from fibers degummed with NaOH solutions of different concentraions (0.1, 0.5, and 1 M) spontaneously formed spherical assemblies (compartments), similarly to native silk fibroin extracted directly from the silkworm silk gland (Supplementary Figure S10a, and Figure S10c–e). However, such assembling behavior has not been observed for RSF obtained from Na_2_CO_3_‐degummed fibers. We further reconfirmed this observation with SAXS analysis and compared the assembling behavior of RSF obtained from NaOH, using Na_2_CO_3_‐based degummed fibers with native silk fibroin taken directly from the silkworm gland; see the Experimental section). The SAXS results, were in agreement with the AFM analysis, pointing to the presence of compartments in RSF from NaOH‐degummed fibers, similar to the native silk, whereas they were absent in the Na_2_CO_3_ degummed fibers, as summarized in Figure 5b,c and Supplementary Figure S11. Furthermore, SAXS analysis confirmed that the radius of the monomeric unit, characterized by a disk shape (designated as “disk” in Figure 5c and Supplementary Figure S11), for RSF obtained from Na_2_CO_3‐_degummed fibers was smaller (3.7 nm) than that of native silk (5.6 nm) and RSF obtained from NaOH‐degummed fibers (~5.3–5.8 nm). We linked both the observations of the small radius of monomeric units and changes in the self‐assembling behavior for RSF obtained from Na_2_CO_3_‐degummed fibers to the loss of light‐chain protein subunits, in agreement with the gel electrophoresis analysis (Figure 5a).

### Rheological Analysis of RSF

Finally, to determine whether different degumming procedures affected the rheological behavior of RSF fluid, we measured changes in viscosity in response to the applied shear, with the shear rates increasing from 0.1 to 500 s^−1^ and decreasing back from 500 to 0.1 s^−1^. We then compared the rheological characteristics of RSF, obtained by different degumming protocols, followed by chemical resolubilization, and native silk fibroin (NSF) extracted directly from the *B. mori* silk gland^[37]^ by dissection (see the Experimental section). The viscosity values recorded for RSF solutions obtained following the protocol that includes the degumming step in the presence of 0.5 and 1 M NaOH were significantly higher than those obtained by the standard degumming protocol (Figure 5 d–e). Such values are comparable to native silk. At the maximum shear rate, (500 s^−1^), the viscosity for silk solutions reconstituted from fibers degummed with 0.5 and 1 M NaOH were 0.03 and 0.05 Pa.s, respectively. However, the viscosity for solutions reconstituted from fibers degummed with 0.1 M NaOH at the 500 s^−1^ shear rate was 0.25 Pa s. At the minimum shear rate (0.1 s^−1^), the viscosity for RSF solutions (the degumming steps with 0.5 and 1 M NaOH were 501.17, and 644.53 Pa s, respectively, and the for 0.1 M NaOH, it was 374 Pa s). In contrast, RSF that was derived from degummed fibers with 0.1 M NaOH were higher than Na_2_CO_3_ (for the 500s–1 shear rate with 0.027 Pa s. and for the 0.1 s^−1^ shear rate the obtained value was 285.5 Pa s), but it was not statistically significant. This observation can be explained by an insufficient concentration of NaOH for effective removal of the gum layer, which further affects the quality of the RSF solution, similarly to Na_2_CO_3_.

## Conclusions

In conclusion, the low temperature, alkiline NaOH‐based treatment for silk fiber degumming developed here improved the fiber mechanics and the thermal stability, which was enabled by preserving the fibroin protein′s molecular integrity, its secondary structure, and the degree of crystallinity in the spun fibers. We have demonstrated that this method also enables obtaining soluble silk of high purity and with structure, as well as self‐assembling behavior and rheological characteristics comparable to those of native silk. Furthermore, we have established a new approach for determining the “true” mechanical characteristics of biological fibers like silk, which accounts for the fibers’ shape asymmetry and the presence of defects. The developed approaches are beneficial for generating silk‐based materials with tailored properties and correctly establishing the mechanical characteristics of asymmetrical fibrous biological materials made of natural and synthetic building blocks.

## Methods

### Degumming of Silk Fibers

Five grams of *B. mori* silkworm silk cocoons (A1 Quality Bombyx mori cocoon from Treeway silk, USA) were cut into small pieces. For the standard degumming treatment, the cut cocoons were boiled for 30 min in an aqueous solution of 0.02 M Na_2_CO_3_ (Cat. S7795, Sigma Aldrich) and rinsed thoroughly with water. For the NaOH degumming treatment (Cat. 001908059100, Biolab), the cut cocoons were rinsed with three different concentrations 0.1, 0.5, or 1 M NaOH. The treatment for 0.1 M was done at 40 °C, and the concentrations for 0.5 and 1 M were carried out at room temperature. After this step, the fibers were rinsed thoroughly with water.

### Resolubilization of Silk Fibroin

The degummed fibers were dissolved in a 9.3 M LiBr (Cat. 013408, Thermo Scientific) solution at 60 °C for 4 h and then dialyzed against distilled water using a dialysis bag of MWCO 3500 for two days. Then, the dissolved centrifuge and insoluble residues were removed.

### Extraction of Silk Fibroin Protein

The fibroin from *Bombyx mori* cocoons was extracted as described.^[24]^ Briefly, silkworm cocoons were chopped and then boiled in a 20 mM sodium carbonate (≥99.5 %, Fischer Chemical, USA) solution at a ratio of 200 mL solution per gram of raw cocoon. The degummed fibers were then washed and dried, followed by desolvation at 60 °C in a concentrated solution of aqueous lithium bromide. The resulting solution was centrifuged and dialyzed against Milli‐Q water.

### Confocal Microscopy Analysis

The 3D images were taken using a Zeiss LSM 800 Confocal Imaging System (Carl Zeiss AG, Germany) with a Plan‐Apochromat 20x/0.8 M27 (FWD=0.55 mm) objective for confocal imaging. At least five 3D images were taken from each of the samples; native silk fibers and different treatments of degummed fibers: Na2CO3, NaOH 0.1 M, NaOH 0.5 M, and NaOH 1 M. Briefly, the samples were incubated with Nile red (with a final concentration of 3 μM) for 16 hours at room temperature and then placed on glass slides and covered and sealed with cover slides. The conditions for the images were excitation with LED lasers of 559 nm (for Nile red excitation) and 346 nm (for intrinsic fluorescence) and the emission of 636 nm (for Nile red) and 442 nm (for intrinsic fluorescence). About 90 slices (~33 μm) of Z‐stacks were taken and the resolution was around 0.105x0.105x0.37 μm/pixel. The 3D images were reconstructed by using Imaris software. In addition, the 3D images were post‐processed using an in‐house developed Matlab script to calculate the cross‐section area and the volume of the fibers.

### Tensile Test

Single fiber specimens for the tensile test were prepared by gluing both the untreated and treated silkworm fibers, produced by *Bombyx mori*, on windowed paper frames with a gauge length of 20 mm. The samples were taken from the middle layers of three different *Bombyx mori* cocoons. Quasi‐static tensile tests of single fibers were conducted with an Instron 5965 universal testing machine (US) equipped with a 10 N load cell at a strain rate of 1 mm/min at room temperature. Fiber clamps were used to hold the paper frame on the instrument, and prior to testing, the side edges of the frame were cut out. A minimum of 17 fibers were tested for each sample, as specified in Table 1, and engineered or “nominal” stress‐strain curves were obtained. The plots were computed in the form of true stress‐true strain curves according to the CSA of each fiber set, as obtained in Figure 2a, and the tensile properties (Young′s modulus (*E*), tensile strength (*σ_u_*), and strain at break (*ε_b_*)) were calculated.

All quantitative mechanical results were expressed in terms of mean ± standard deviation (SD).

### Bulk FT‐IR Spectral Measurements

FT‐IR spectra for the bulk regenerated fibroin were obtained using a Nicolet iS50 FT‐IR spectrometer equipped with an ATR Smart iTX (attenuated total reflectance) accessory with a resolution of 4 cm‐1 and 32 individual scans for each measurement. At least three replicates were measured for each sample.

At least three measurements were taken of each sample, and the obtained spectra were normalized and averaged as described in the Methods section. Seven peaks were selected for the fitting analysis: an intermolecular *β*‐sheet (1609, 1621, and 1631 cm^−1^), *α*‐helix/ random coil (1650 cm^−1^), *β*‐turn (1673 cm^−1^), and an antiparallel amyloid *β*‐sheet (1695 and 1703 cm^−1^) as described in [70].

### Analysis of IR Spectra

All the IR spectra (bulk FT‐IR and nano IR) between ~1720 and 1600 cm^−1^ were linearly baselined to cover the amide I region. To resolve the secondary structures of the samples, the spectra were normalized and the replicate spectra from each sample were averaged. Then the spectra were fitted (by OriginPro 2019b 64bit software) by selecting seven Gaussian peaks (1609, 1621, 1631, 1650, 1673, 1695, and 1703 cm^−1^ with a freedom of 2 cm^−1^) based on [70]. The fitting analysis for all the spectra was carried out to fit the converged and Chi‐Sqr tolerance value of 1E‐6. The secondary structures’ interpretations of these peaks were 1609, 1621, and 1631 cm^−1^ for intermolecular *β*‐sheet, 1650 cm^−1^ for *α*‐helix and random coil, 1673 cm^−1^ for *β*‐turn, and 1695 and 1703 cm^−1^ for the antiparallel amyloid *β*‐sheet.

### High‐Resolution Scanning Electron Microscopy (HRSEM) Analysis

HRSEM images were obtained using Ultra‐55 and SIGMA Ultra‐high‐resolution SEM (Carl Zeiss, Germany). The samples were placed onto aluminum stubs and fixed with carbon tape.

### X‐Ray Diffraction (XRD) Spectroscopy

XRD of crystalline structures associated with silk I and silk II polymorphisms present in silk fibroin fibers was carried out in reflection geometry using a TTRAX III (Rigaku, Japan) theta‐theta diffractometer with a rotating Cu anode operating at 50 keV and 200 mA. A bent graphite monochromator and a PMT detector were aligned in the diffracted beam and *θ*/2*θ* scans were performed under specular conditions in the Bragg‐Brentano mode with variable slits. The 2*θ* scanning range was 1–80 degrees with a step size of 0.025 degrees and a scan speed of 0.4 degrees per minute.

### Atomic Force Microscopy (AFM)

A silk sample drop (50 μl) was placed on freshly cleaved mica and incubated at RT for 5 min. The excess fluid was removed by carefully applying filter paper to the edge of the drop. The mica was washed 3–4 times with water (1 ml in each wash) and dried with a nitrogen stream. Next, the sample was imaged on an AFM (JPK Nano wizard 4 AFM (JPK, Germany)), with a pixel resolution of 1024× 1024 (for images with a size of 5 μm × 5 μm) using AC240 or AC160 cantilevers (Olympus) in AC mode at 23 °C and 35 % humidity.

### Dynamic Mechanical Analysis (DMA)

The DMA tests were performed on a Discovery 850 (TA instrument) under a multi‐frequency strain mode. The standard polymer test parameters were used: (i) the temperature ramp rate was at 3 °C/min, (ii) the frequency was at 1 Hz, and (iii) a stress control of 3× 10^−3^ N. These parameters were selected based on other research on silk.^[71]^ A preload force equivalent to 0.012 N was applied to keep the testing fiber in tension throughout the dynamic oscillation. Note that temperature scans are only shown with increasing temperature. The DMA procedures were on a full‐range temperature scan from 27 to +270 °C.

### Gel Electrophoresis (SDS‐PAGE)

First, 25 μg of the silk fibroin sample were loaded and run on a gradient gel (4–20 %) from Geba using the manufacturer′s protocol. The gel was stained overnight with InstantBlue® Coomassie Protein Stain (ISB1 L) (ab119211) and washed for several hours with water.

### Silkworm Dissection

*Bombyx mori* larvae at their fifth instar were anesthetized with N2 for 15 min and then rapidly dissected by removing the head and applying a longitudinal dorsal incision. The silk glands were gently extracted and rinsed with Mili‐Q water and then gently placed on a glass slide (25×16 mm), which was then set on a microscope stage for microscopy detection.

Small‐Angle X‐Ray Scattering (SAXS) Measurements

Solution SAXS measurements were performed at ID02 beamline at the European Synchrotron Radiation Facility (ESRF), using a beam size of $32.4\times 145\;\;\mu m^{2}$ (vertical and horizontal, respectively), a photon energy of 12.23 keV, an Eiger2 4 M (Dectris AG) detector, a sample‐to‐detector distance of 3.114 m, and an exposure time of 0.1 s.^[72]^ SAXS models were computed by X+ and D+ software using a water electron density of $333\;e\cdot mm^{-3}$ .[^73^, ^74^, ^75^, ^76^]

We fitted the data to a linear combination of uniform disks, and either sphere and/or slab geometries.^[77]^ We took into account the polydispersity in the radii as explained.[^75^, ^76^]

Based on the contribution of the intensities of each model at the smallest scattering vectors and the volume of each model, we computed the mass fraction of disks, spheres, and slabs (Figure 5b,c).

### Rheological Analysis

Rheological characterization was carried out using a HR‐20 Discovery Hybrid Rheometer (TA Instruments, US) using an aluminum 40 mm diameter parallel plate geometry at 25 °C. The geometry was lowered to a gap of 100 μm at the slowest speed possible. A small amount of distilled water was applied around the specimen and the area was enclosed using a loose‐fitting cover, to avoid drying and skin formation. The sample was initially sheared at a constant shear rate of 1 s^−1^ for 100 s to evenly distribute the liquid and to remove any residual stresses due to the previous handling of the sample. Next, an oscillation frequency test was carried out with a strain of 0.02 (within the material′s linear elastic region) and an angular frequency of 100 rad/s to 0.1 rad/s. Then, the samples were subjected to two repetitive steps of flow sweep tests, each consisting of an increase from 0.1 to 500 s^−1^ and a decrease from 500 to 0.1 s^−1^. The last step included a second oscillation frequency test with the same parameters as before.

### Statistical Analysis

One‐way analysis of variance (ANOVA) was conducted using OriginPro 2022 software to determine whether significant differences existed among the mean values of the experimental groups. A difference among groups was statistically significant at p<0.05. “ns” stands for “no statistical difference”.

## Conflict of Interests

The authors declare no competing interests.

1

## Supporting information

As a service to our authors and readers, this journal provides supporting information supplied by the authors. Such materials are peer reviewed and may be re‐organized for online delivery, but are not copy‐edited or typeset. Technical support issues arising from supporting information (other than missing files) should be addressed to the authors.

Supporting Information

## Data Availability

The datasets generated and analyzed during the current study are available from the corresponding author on reasonable request.
