# Supplementary material for: Fine Structural Analysis of Degummed Fibroin Fibers Reveals Its Superior Mechanical Capabilities
Source: ChemSusChem. 2024 Sep 25;18(1):e202401148. doi: 10.1002/cssc.202401148 (PMC11696198; doi:10.1002/cssc.202401148)
Supplement: Supplementary file 1 — Supporting Information [file CSSC-18-e202401148-s001.pdf]

ChemSusChem

Supporting Information

Fine Structural Analysis of Degummed Fibroin Fibers Reveals Its Superior Mechanical Capabilities

D. Eliaz, I. Kellersztein, M. E. Miali, D. Benyamin, O. Brookstein, C. Daraio, H. D. Wagner, U. Raviv, and U. Shimanovich*

Fine structural analysis of degummed fibroin fibers reveals its superior mechanical capabilities

D. Eliaz[1,$,#], I. Kellersztein[2#], M.E. Miali[1], D. Benyamin[3], O. Brookstein[1], C. Daraio[2], H. D. Wagner[1], U. Raviv[3] and U. Shimanovich[1]*

[1]Department of Molecular Chemistry and Materials Science, Faculty of Chemistry, Weizmann Institute of Science, 7610001 Rehovot, Israel
[2]Division of Engineering and Applied Science, California Institute of Technology, Pasadena, CA 91125, USA
[3]Institute of Chemistry, The Hebrew University of Jerusalem, Edmond J. Safra Campus, Givat Ram, Jerusalem 9190401, Israel

[#] These authors contributed equally to this work.
[$] present address: SilkIt Ltd. Ness Ziona 7403626, Israel

* To whom correspondence should be addressed: ulyana.shimanovich@weizmann.ac.il.

Supplementary Information

Supplementary Table S1: *Summary of the degumming conditions and efficiency of the sericin removal.*

Sample	Cocoon weight (mg)	Concentration (M)	Incubation (min)	Temperature (°C)	Dry weight (mg)	Dry wet weight ratio	Sericin removal (%)
Na_2CO_3	500	0.02	30	100	362.5	0.72	27.5
NaOH		1	10	Room temp.	364.1	0.73	27.2
		0.5	15	Room temp.	356.1	0.71	28.8
		0.1	30	40	369.9	0.74	26.0

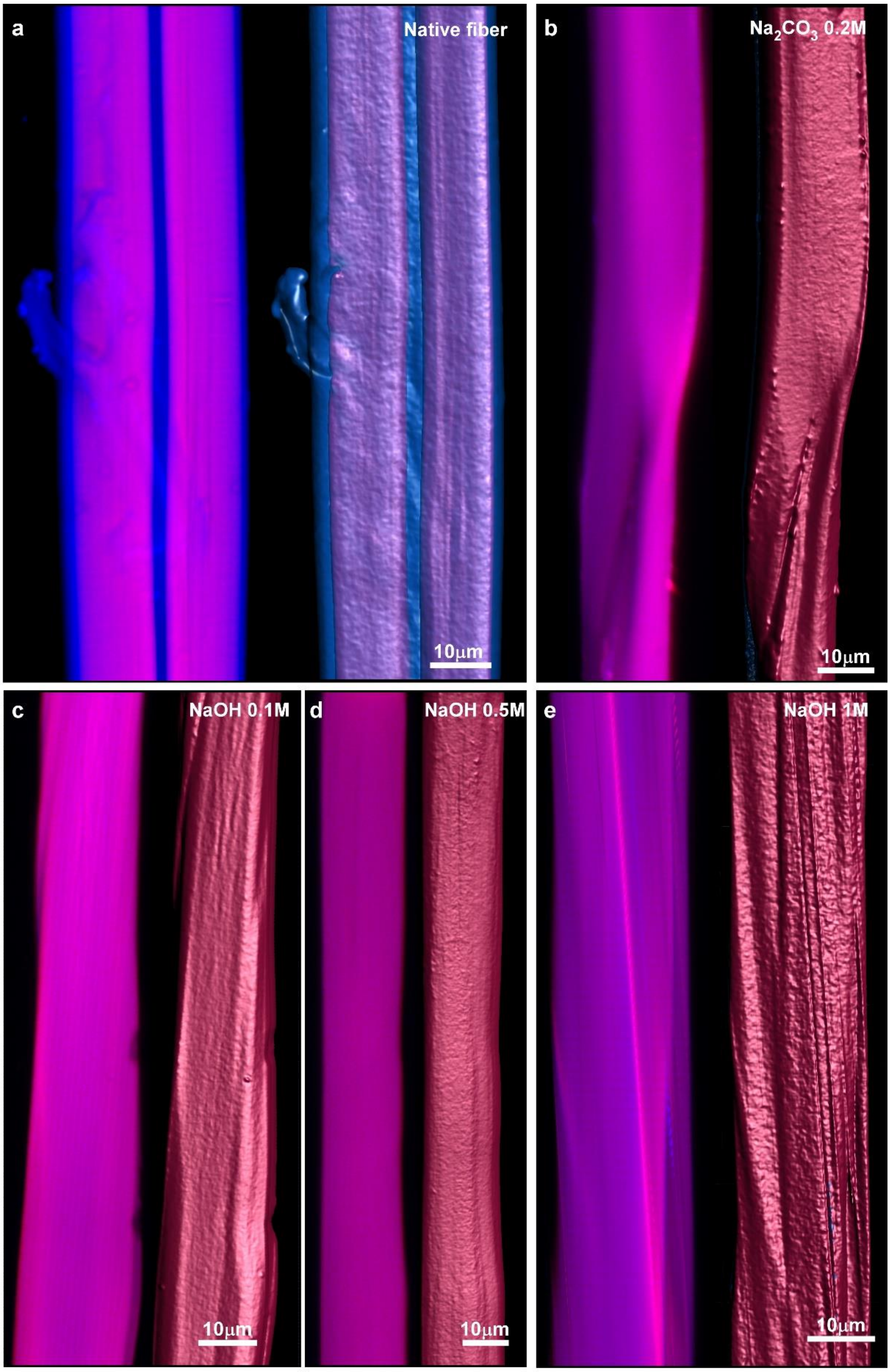

Supplementary Figure S1: Confocal microscopy analysis of degummed RSF fibers obtained by using different degumming approaches. *The confocal images depicting higher length of the fibers shown in figure 2 of the main text. Confocal images of the untreated* ***(a)*** *and deggumed fibers* ***(b)*** *Na_2CO_3,* ***(c)*** *NaOH 0.1M,* ***(d)*** *NaOH 0.5M and* ***(e)*** *NaOH 1M). The fibers stained with Nile Red dye, where at the left side is longitudinal 3D confocal image, and in the right side is longitudinal 3D confocal processed image. Scale bars are 10 µm.*

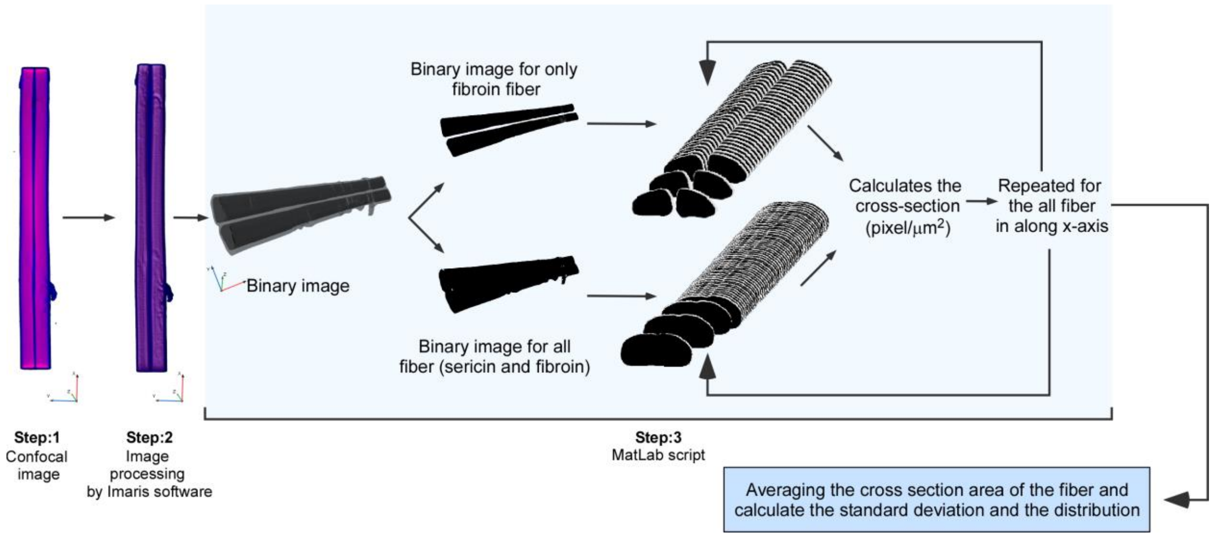

Supplementary Figure S2: Algorithm describing the process of the cross-sectional area analysis performed by using Matlab script*. Step 1: acquisition of 3D confocal images; Step 2: images post-processing by using Imaris image analysis software; Step 3: Conversion of the confocal two channels images into binary images by using a home written script, followed by collection of the cross-sectional area for each pixel along the silk fiber.*

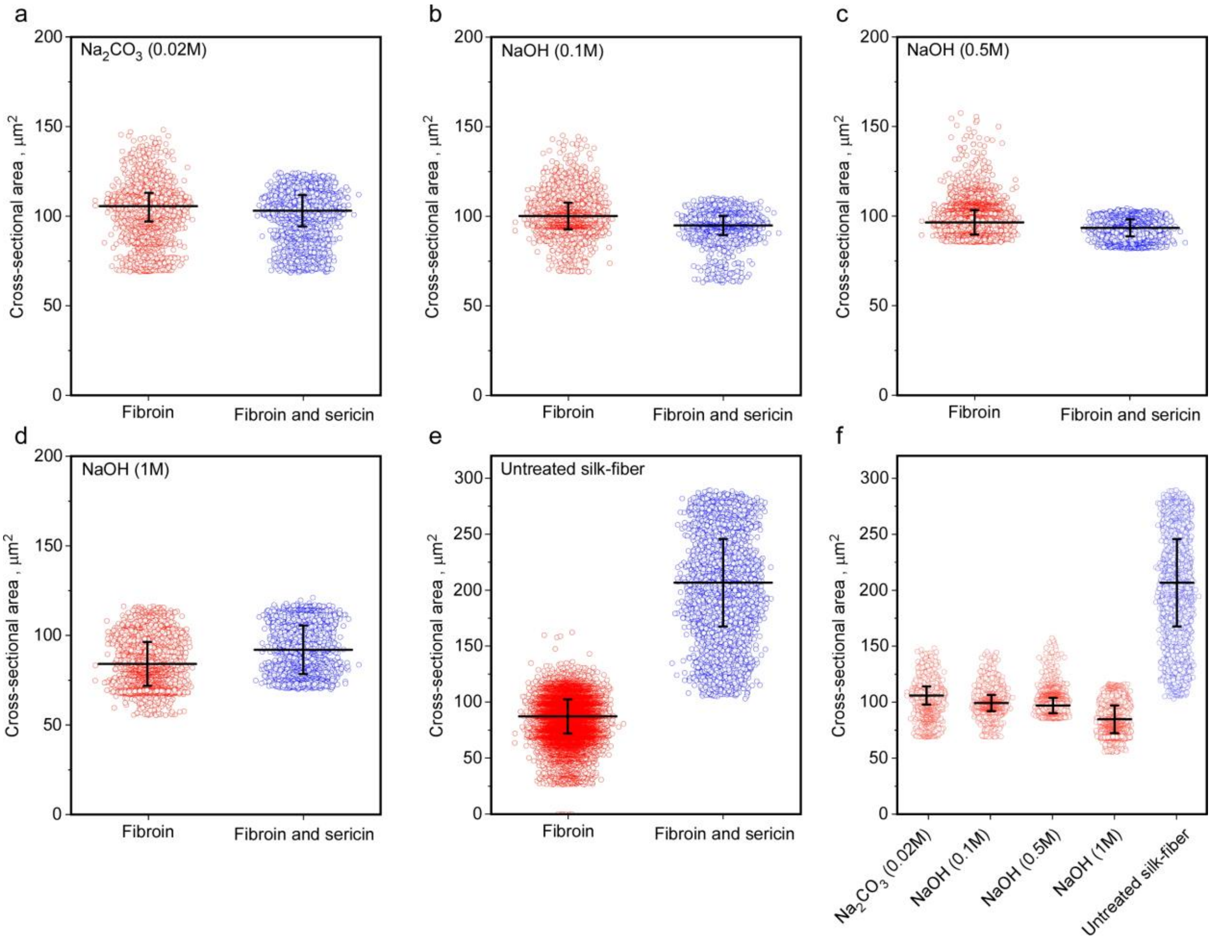

Supplementary Figure S3: Computational analysis of fiber silk cross-sectional area. *(a-e) Cross-sectional area results obtained from the computational analysis for degummed fibers and untreated silk-fibers. The red dots represent the cross section areas obtained from fibroin only component (stained with Nile red) and the blue dots correspond to areas obtained from fibroin and sericin components (intrinsic fluorescence). The statistical information is presented in Supplementary Table S2. (f) Comparison between all types of fibers.*

Supplementary Table S2: *Summary of the data used for analysis of the crossectional area, based on the confocal images, of the fibers degummed using different degumming procedures (Supplementary Figure S3).*

Sample	Fluorescence signal	Number of cross-section	Mean (μm^2)	Standard Deviation (μm^2)	Median (μm^2)
Untreated silk-fiber	Nile red (635 nm)	41443	86.6	15.2	88.2
	Intrinsic fluorescence (443 nm)	25596	206.3	38.9	199.6
Na2CO3 (0.02M)	Nile red (635 nm)	11766	105.3	8	106.7
	Intrinsic fluorescence (443 nm)	11766	103.2	8.6	103.1
NaOH (0.1M)	Nile red (635 nm)	11924	100.2	7.4	98.7
	Intrinsic fluorescence (443 nm)	11924	95	5.4	95.7
NaOH (0.5M)	Nile red (635 nm)	11762	96.3	6.7	95.4
	Intrinsic fluorescence (443 nm)	11762	93.2	4.7	92.7
NaOH (1M)	Nile red (635 nm)	11552	84	12.4	82
	Intrinsic fluorescence (443 nm)	11552	91.8	13.5	86.5

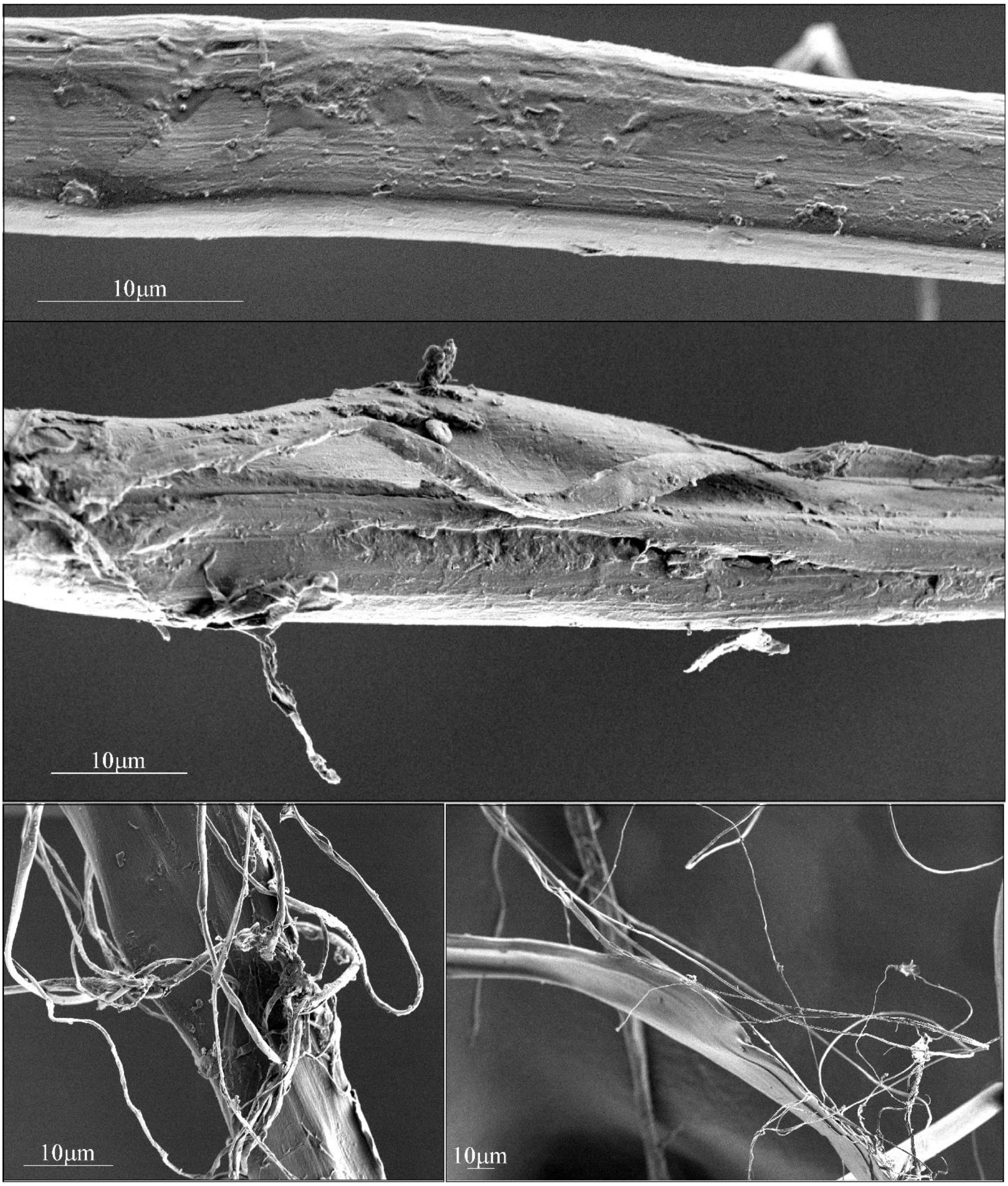

Supplementary Figure S4: *SEM images showing that treating the silk fibroin fibers with 1M NaOH disintegrated the microscale fiber into separated nanofibrils.*

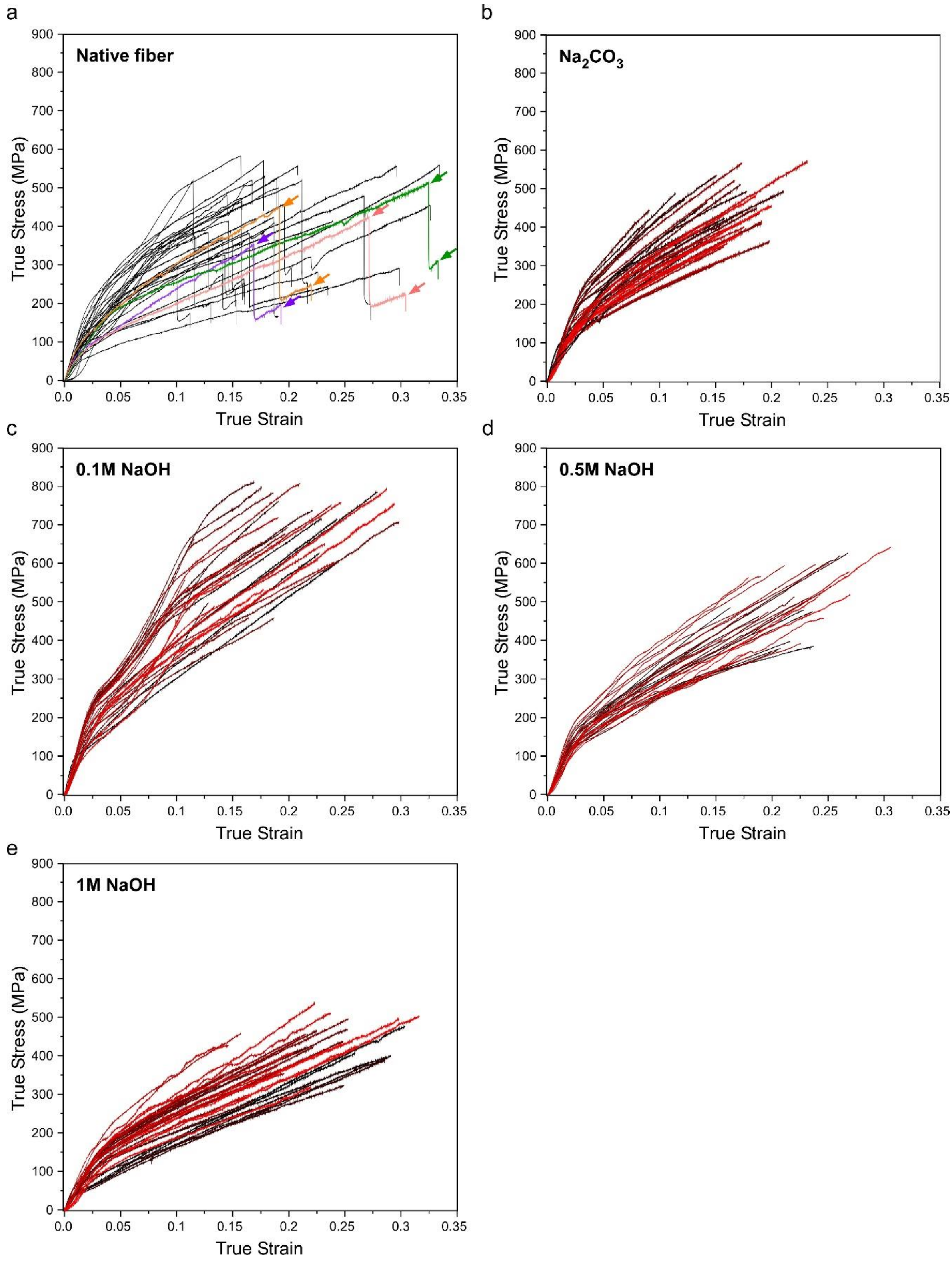

Supplementary Figure S5: True stress- true strain curves of (a) *untreated silk-fiber;* ***(b)*** *Na_2CO_3 treated silk-fiber;* ***(c)*** *0.1M NaOH treated silk-fiber;* ***(d)*** *0.5M NaOH treated silk-fiber; and* ***(e)*** *1M NaOH treated silk-fiber.*

Supplementary note S1

Determination of the true stress and true strain values of the fibers: The engineering or "nominal" stress and strain values, denoted here as σ_e and ε_e respectively, are obtained from the measured load (P) and displacement (δ) values according to the following relations:

$$\sigma_e = \frac{P}{A_0} \tag{E1}$$

$$\varepsilon_e = \frac{\delta}{l_o} \tag{E2}$$

where A_0 is the initial cross-section area of the sample and considered to be constant throughout the test, and l_0 is the initial length of the sample, equivalent to the gauge length of the specimen in the tensile test. When the stress (σ_e) is plotted as a function of strain (ε_e), an engineering stress-strain curve is obtained. This curve should be interpreted with caution, especially beyond the elastic limit, as the sample dimensions experience a substantial change from their initial values.

To elucidate a more accurate mechanical behavior under tensile stresses of the fibers, one can use the engineering stress and strain to compute the true stress (σ_t) and true strain (ε_t) of the silk fibers. The mathematical calculation of the true stress and true strain is possible under the assumption of volume consistency during stretching, which has been confirmed by Guinea et al. [1] for silk fibers. This assumption is valid in the elastic region of the curve because any changes in volume in this region will be very small. Similarly, the assumption is valid in the plastic region because materials, such as silk fibers, are considered to be incompressible during plastic deformation. Furthermore, the true stress and true strain can be mathematically defined as:

$$\sigma_t = \frac{P}{A} \tag{E3}$$

$$d\varepsilon_t = \frac{dl}{l} \tag{E4}$$

where A is the cross-sectional area of the sample, $d\varepsilon$ is the increment of strain, dl is the increment in length and l is the length of the fiber. Considering that the volume of the sample remains constant:

$$A_0 l_0 = Al \rightarrow A = \frac{A_0 l_0}{l}$$

$$\sigma_t = \frac{Pl}{A_0 l_0} = \sigma_e(1 + \varepsilon_e) \quad \text{(E5)}$$

$$\varepsilon_t = \int_{l_0}^{l} \frac{dl}{l} = ln\left(\frac{l}{l_o}\right) = ln(1 + \varepsilon_e) \quad \text{(E6)}$$

Supplementary note S2

Statistical distribution to calculate the strength of the fibers: When applying stress to a stiff material, stress concentrations at local areas emerge because of changes in specimen geometry, cracks, and surface irregularities, among others. All these manifestations of stress concentrations are observed in silk fibers, where their geometry is constantly changing as formed by the *B.mori*, and the irregular sericin layer presents micro-cracks along the fiber surface, as shown in Figure 3. The given results of tensile strength on such fibers differ because of dissimilarities in the samples, which can be attributed to defects, and therefore, the strength of silk fibers in the present case cannot be defined by a single value. A statistical distribution is then required to quantitively address the dependence of the strength of the fibers on their flaw distribution.

In this regard, the Weibull distribution has been usually considered the most suitable statistical model as the distribution considers the lowest possible fracture strength of zero (i.e., the distribution is bounded), it yields a simple and useful graphical plot, it provides reasonably accurate failure approximations even with a small population of samples, and finally, the parameters of the distribution allow to account shape assymetry[2–4]. To report the strength distribution of a single fiber, the two-parameter Weibull distribution, or Weibull cumulative distribution function, is often used:

$$f(\sigma) = 1 - e^{(-(\sigma/\alpha)^{\beta})} \quad \text{(E7)}$$

where f(σ) is the probability of failure of the fiber under applied stress (σ), α is the scale parameter of the distribution, which represents the average or characteristic strength of the fiber according to the distribution, and it depends on the stress configuration and test specimen size,

and β is the shape parameter, which depends on the defect sizes, and is a measure of the variability of the strength (higher β values mean lower strength variability) and hence the fiber reliability. To calculate the scale (α) and shape (β) parameters of the distribution a double logarithm of the Weibull equation can be applied as follows:

$$\ln\left(1 - f(\sigma)\right) = -\left(\frac{\sigma}{\alpha}\right)^{\beta} \quad \text{(E8)}$$

$$\ln\left[-\ln\left(1 - f(\sigma)\right)\right] = \beta \ln \sigma - \beta \ln \alpha \quad \text{(E9)}$$

Then, eq. (E9) is plotted yielding a linear graph of slope β. The Weibull scale parameter (α) of the fiber is readily obtained from the intercept of this line, given by:

$$\alpha = e^{\left(intercept/_{\beta}\right)} \quad \text{(E10)}$$

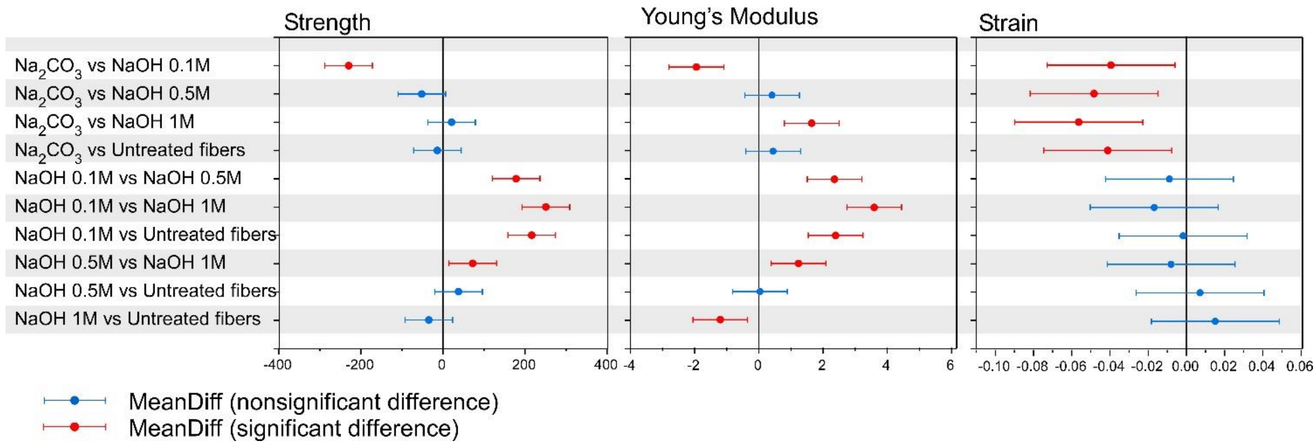

Supplementary Figure S6: Statistical analysis for the mechanical measurements. *To determine whether significant differences existed among the mean values, one-way analysis of variance (ANOVA) was conducted between all the samples. A difference among groups was statistically significant at* $p<0.05$.

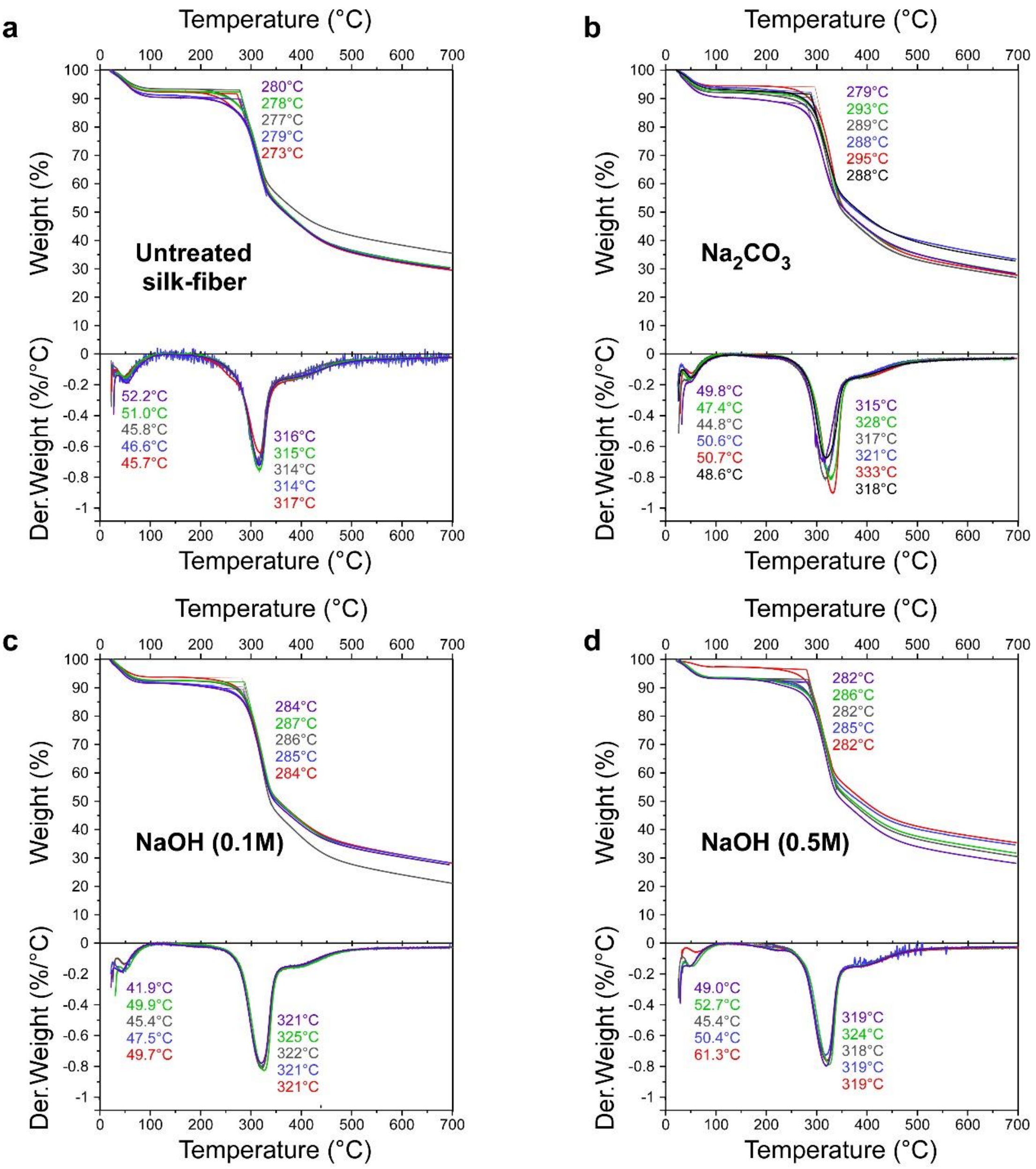

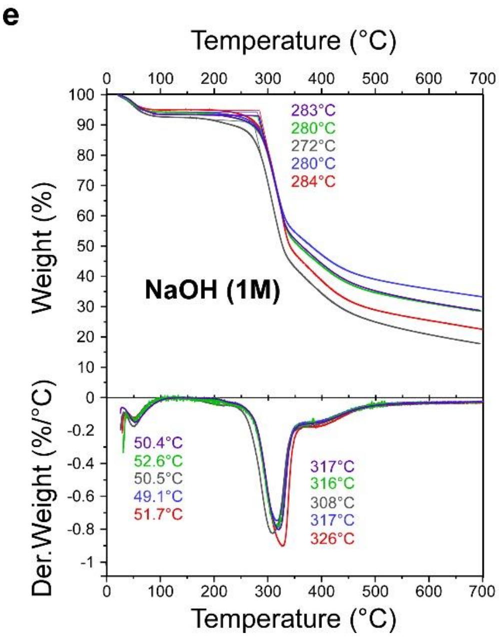

Supplementary Figure S7:
The effects of the degumming procedures on the thermal stability of the silk fibers. *Five independent reports of thermogravimetric analysis (TGA) were performed for each sample of untreated silk-fiber **(a)**, Na_2CO_3 **(b)**, NaOH 0.1M **(c)**, NaOH 0.5M **(d)** and NaOH 1M **(e)**. The graphs demonstrate weight (%)versus temperature (°C) at the upper panel and the onset calculation appears for each measurement. The bottom panels present the derivative for these measurements and the calculation for water loss and the maximum degradation next to the peaks.*

Supplementary Table S3: Analysis of weight loss based on the results obtained from TGA measurements.

	**untreated**	**Na_2CO_3**	**NaOH 0.1M**	**NaOH 0.5M**	**NaOH 1M**
Weight of water loss I (%)	7.9	6.4	7.3	6.4	6.1
STD. (%)	1.1	1.8	0.8	0.1	0.8
Weight loss II (%)	39.5	43.5	45.7	41.6	44.2
STD. (%)	1.8	2.4	1.8	2.9	4.1
Weight loss III (%)	21.3	20.0	20.2	19.5	21.5
STD. (%)	0.9	1.2	1.1	0.7	1.8
Weight remain (%)	31.1	29.8	26.5	32.2	26.0
STD. (%)	2.4	2.6	2.9	3.1	6.1

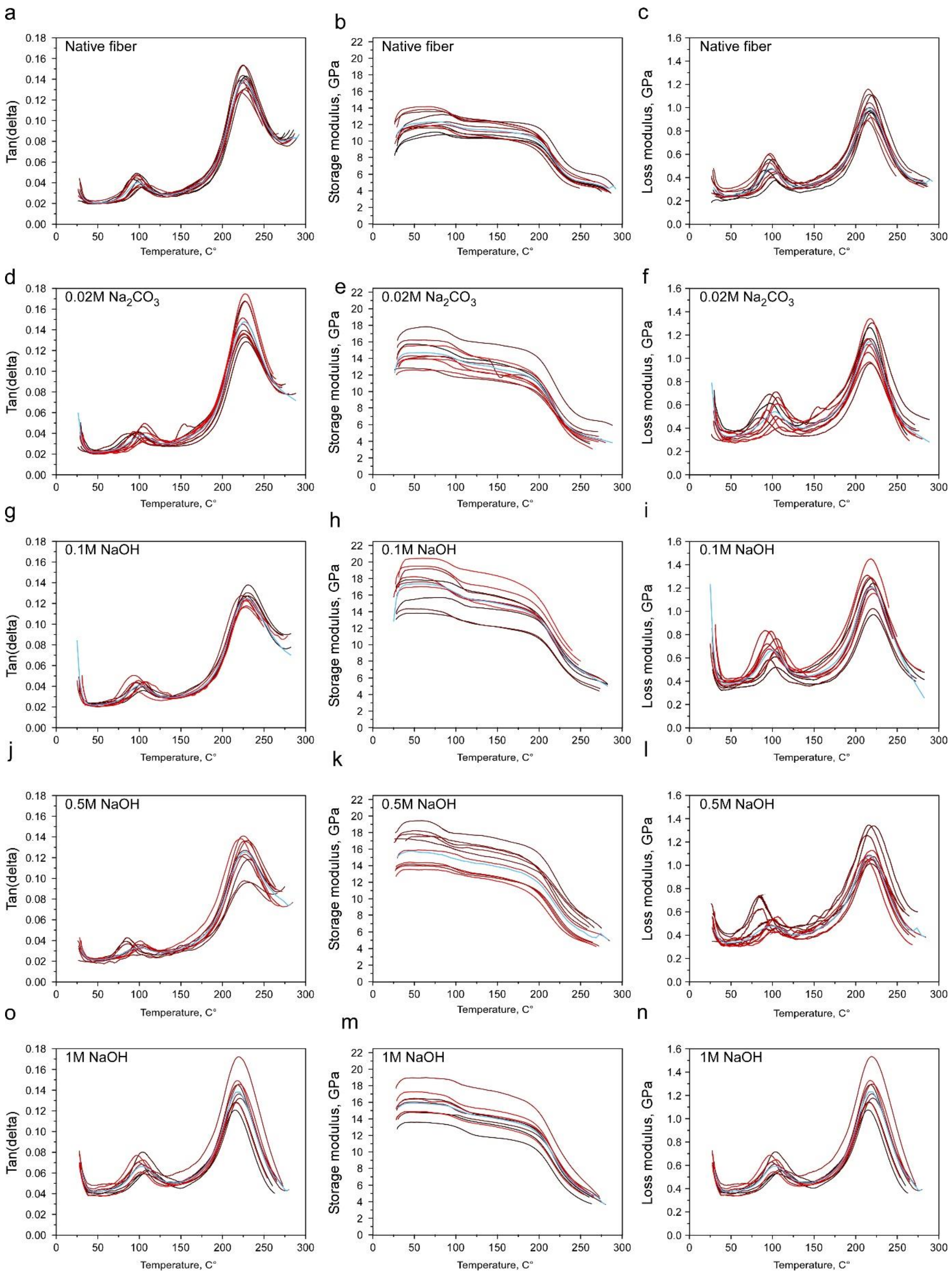

Supplementary Figure S8: DMA analysis. *The tang (delta) graphs (**a,d,g,j** and **o**), the storage modulus graphs (**b,e,h,k** and **m**) and the loss modulus graphs (c,f,I,l and n) presented for different degumming procedures. The degumming procedures are indicated at the top left corner of each graph. The averaged graphs lines for each sample are shown in blue.*

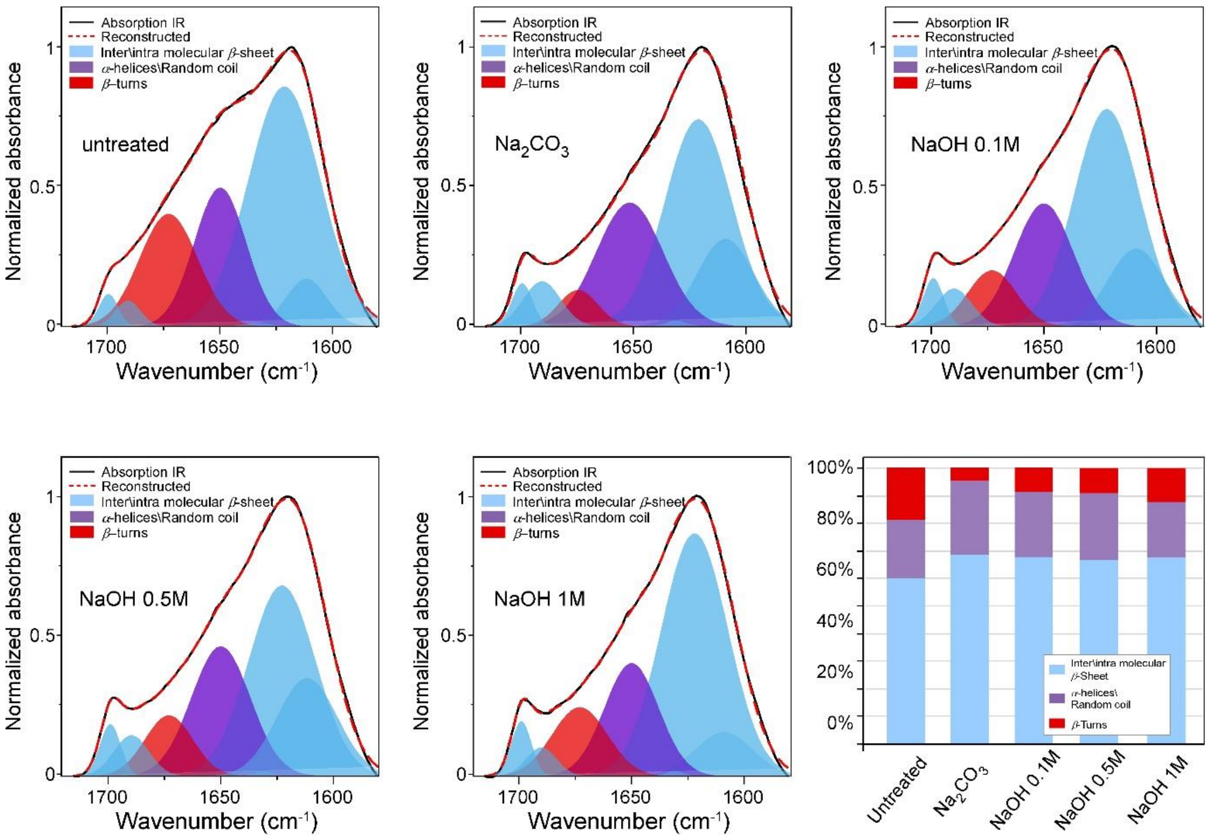

Supplementary Figure S9: Fourier Transform Infrared spectroscopy (FTIR) analysis of silk fibers, degummed and untreated. *FTIR spectra of untreated silk fibers untreated **(a)**, fibers degummed by using Na_2CO_3 **(b)**, NaOH 0.1M **(c)**, NaOH 0.5M **(d)**, and NaOH 1M **(e)**. **(f)** Bar chart of the relative amounts of the β-sheets at 1610−1635 cm^{-1}, the anti-parallel β-sheets at 1690−1705 cm^{-1}, random coil and α-helixes at 1635−1665 cm^{-1}, and β-turns at 1665−1690 cm^{-1}.*

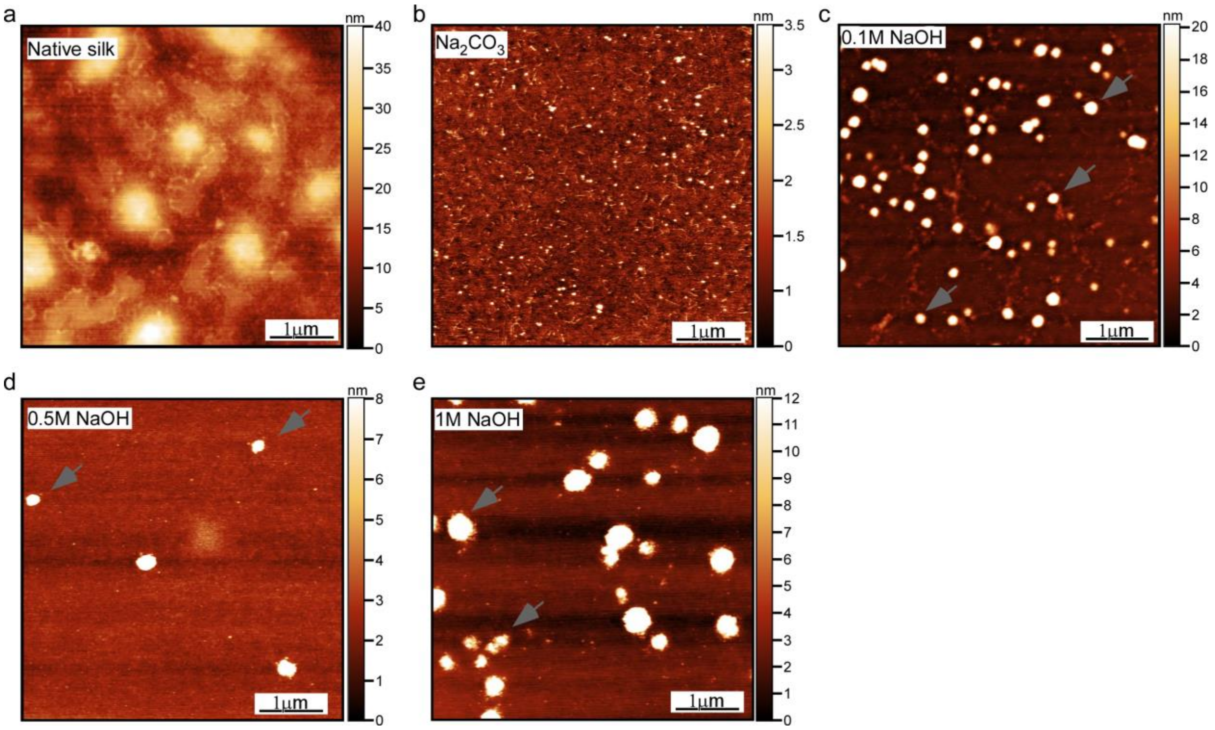

Supplementary Figure S10: *Atomic force microscopy (AFM) analysis of the native silk fibroin (a) extracted directly from the B.mori silkworm silk gland via dissection (see Experimental section) and (b)-(e) of the resolubilized silk fibroin from the silk fibers initially degummed under different conditions: in the presence of Na_2CO_3* ***(b)****, NaOH 0.1M* ***(c)****, NaOH 0.5M* ***(d)****, and NaOH 1M* ***(e)****.*

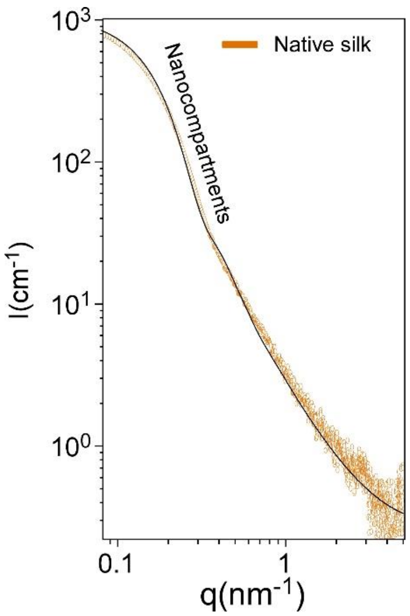

Supplementary Figure S11: *SAXS analysis of native soluble silk fibroin, extracted directly from the silkworm silk gland, confirming the presence of spherical assemblies (termed nanocompartments) and shown in the graph. Supplementary Table S4 summarizes the parameters of models used to fit the SAXS data shown in Supplementary Figure S11 and Figure 5b of the main text.*

Supplementary Table S4. *The parameters of the models used in the SAXS analysis (Supplementary Figure S11 and Figure 5b of the main text). R is the radius (of the sphere or the disk). In both models we assumed polydispersity with a Gaussian distribution of radii whose variance was* σ^2.

	Model	R[nm]	σ^2[nm^2]	Hight [nm]
NSF	Disk	5.6	1	0.58
NSF	Sphere	10.8	3	-

REFERENCES

(1) Guinea, G. V.; Pérez-Rigueiro, J.; Plaza, G. R.; Elices, M. Volume Constancy during Stretching of Spider Silk. *Biomacromolecules* **2006**, *7* (7), 2173–2177. https://doi.org/10.1021/bm060138v.

(2) Quinn, J. B.; Quinn, G. D. A Practical and Systematic Review of Weibull Statistics for Reporting Strengths of Dental Materials. *Dent. Mater.* **2010**, *26* (2), 135–147. https://doi.org/10.1016/J.DENTAL.2009.09.006.

(3) Newell, J. A.; Kurzeja, T.; Spence, M.; Lynch, M. Analysis of Recoil Compressive Failure in High Performance Polymers Using Two and Four Parameter Weibull Models. *High Perform. Polym.* **2002**, *14* (4), 425–434. https://doi.org/10.1177/095400830201400408.

(4) Wagner, H. D.; Phoenix, S. L.; Schwartz, P. A Study of Statistical Variability in the Strength of Single Aramid Filaments. *J. Compos. Mater.* **1984**, *18* (4). https://doi.org/10.1177/002199838401800402.
